# Supplementary material for: Shikonin Reduces Growth of Docetaxel-Resistant Prostate Cancer Cells Mainly through Necroptosis
Source: Cancers (Basel). 2021 Feb 20;13(4):882. doi: 10.3390/cancers13040882 (PMC7923752; doi:10.3390/cancers13040882)
Supplement: Supplementary file 1 [file cancers-13-00882-s001.pdf]

PC3 – p21

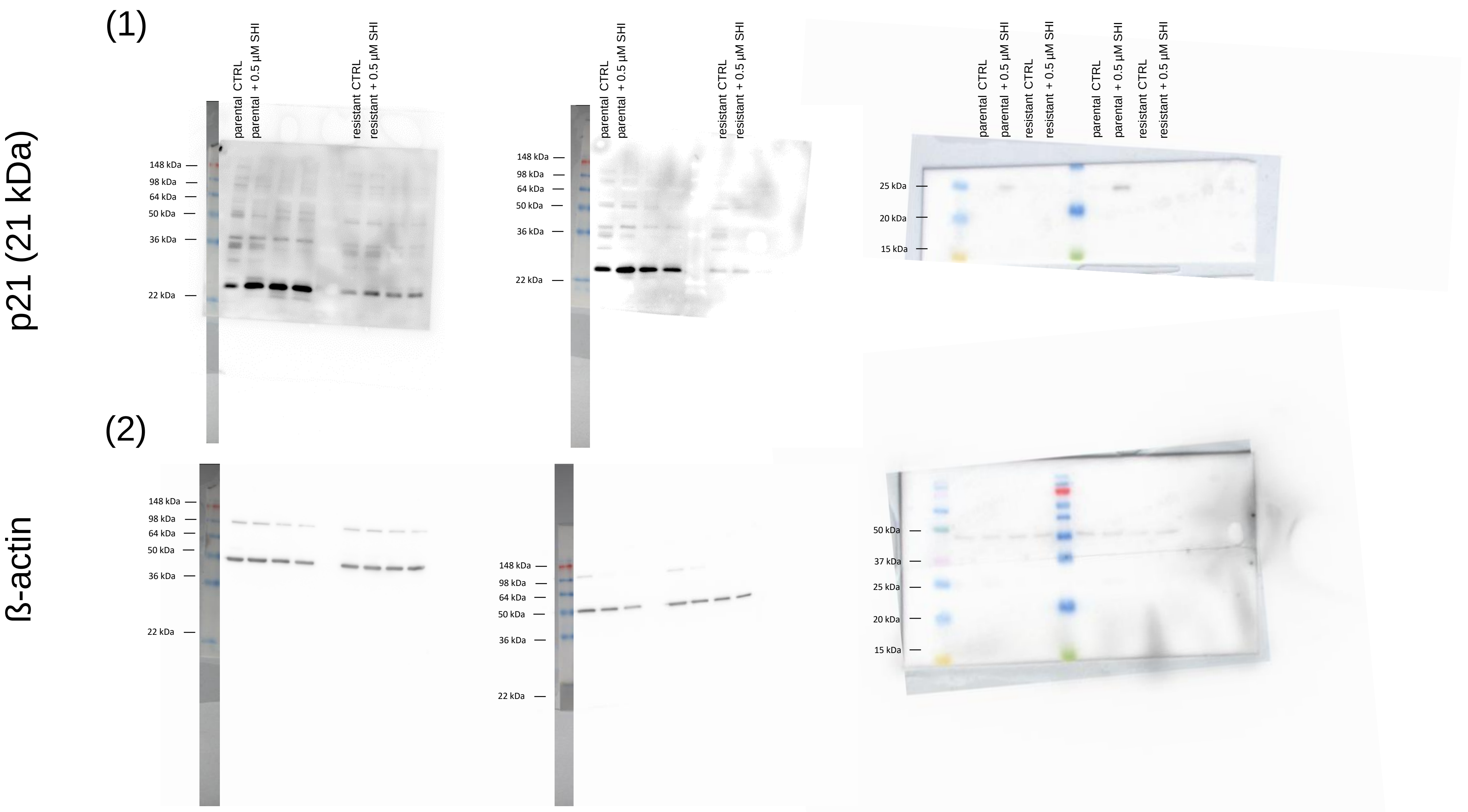

Figure S1a: Detailed information about Figure 5 - Protein expression profile of cell cycle regulating proteins in parental and DX-resistant PC3 cells. Protein expression of p21 (1), corresponding protein expression of ß-actin (2).

PC3 – p27

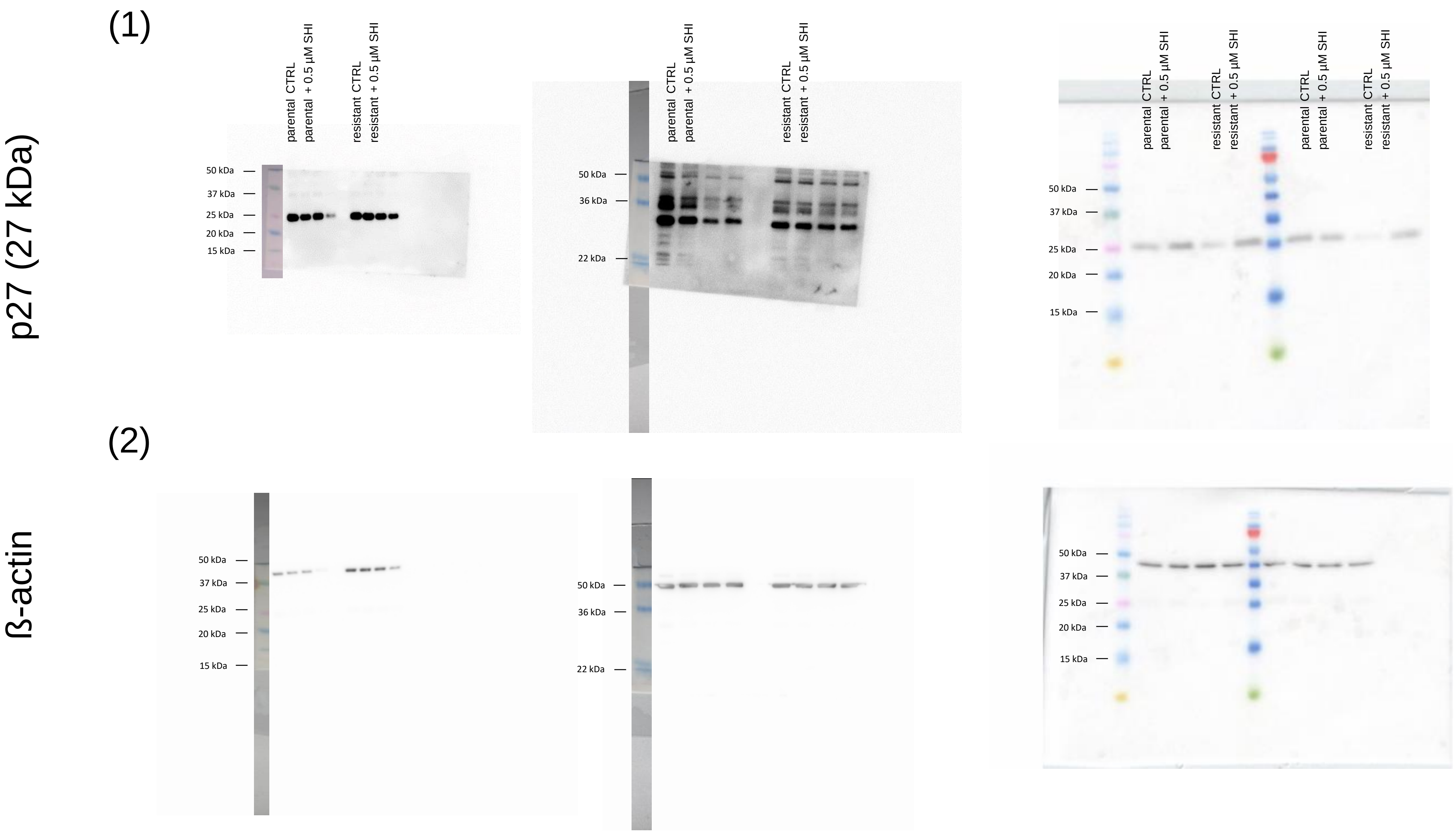

Figure S1b: Detailed information about Figure 5 - Protein expression profile of cell cycle regulating proteins in parental and DX-resistant PC3 cells. Protein expression of p27 (1), corresponding protein expression of ß-actin (2).

PC3 – Cyclin A

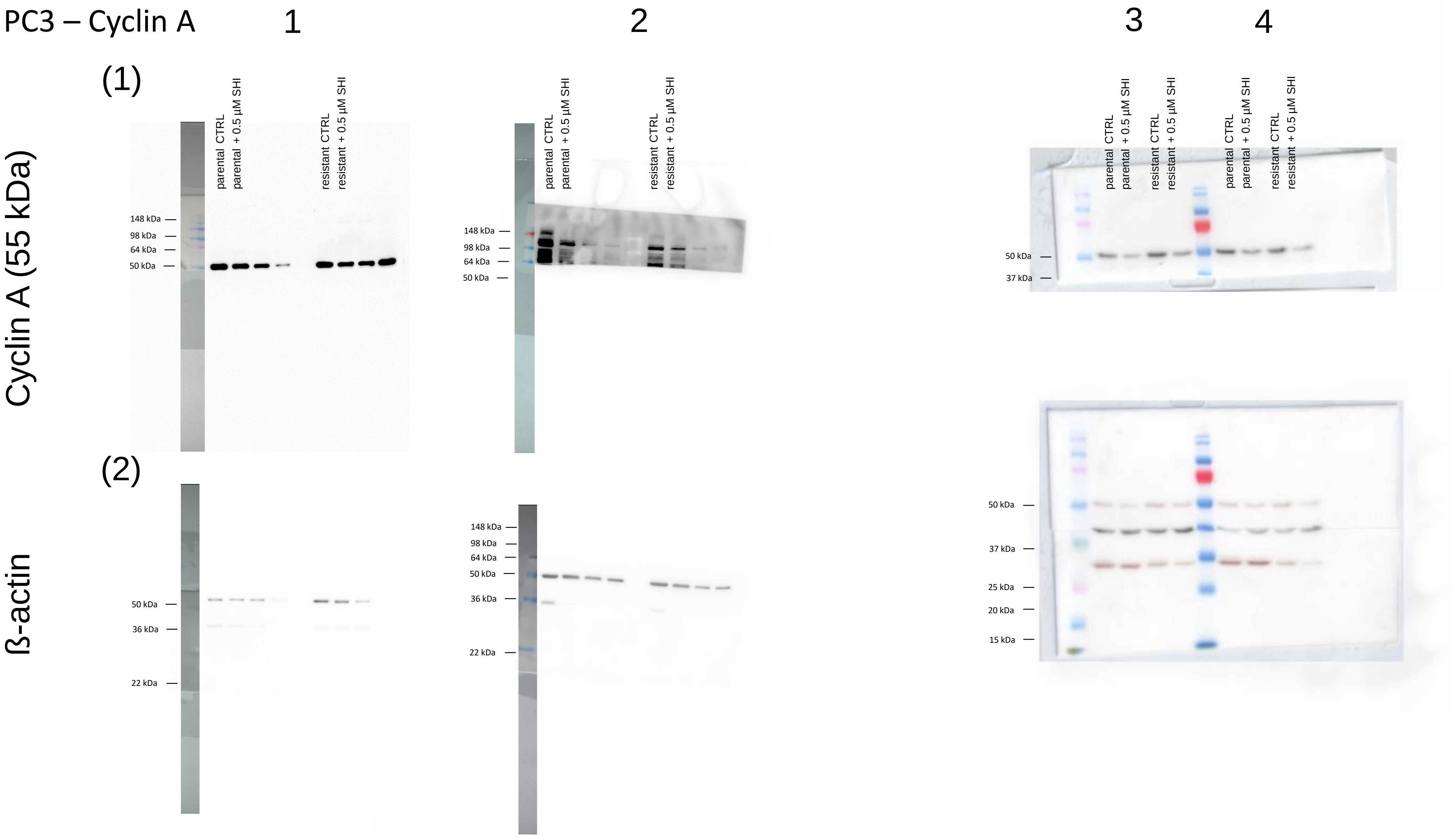

Figure S1c: Detailed information about Figure 5 - Protein expression profile of cell cycle regulating proteins in parental and DX-resistant PC3 cells. Protein expression of Cyclin A (1), corresponding protein expression of ß-actin (2).

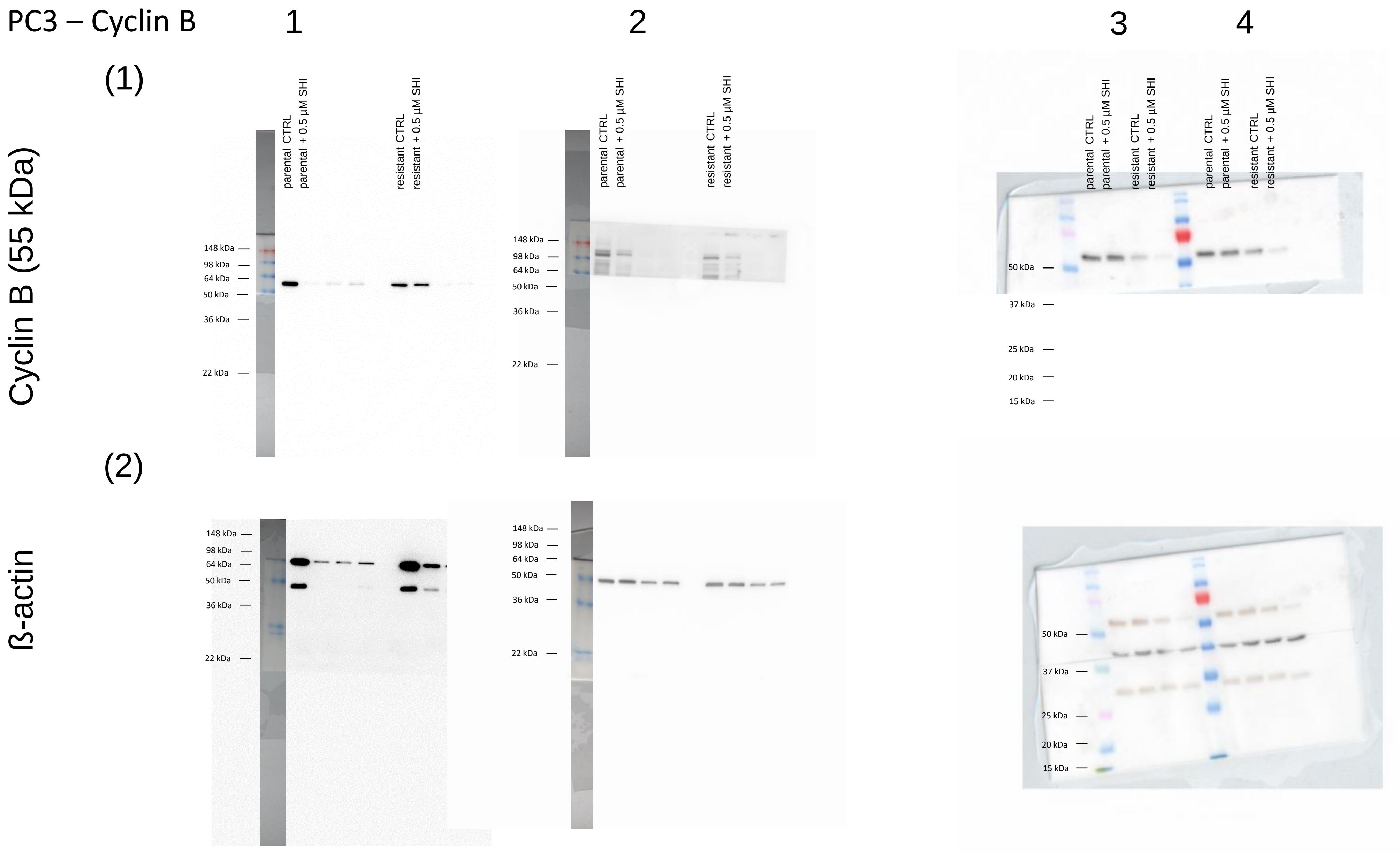

Figure S1d: Detailed information about Figure 5 - Protein expression profile of cell cycle regulating proteins in parental and DX-resistant PC3 cells. Protein expression of Cyclin B (1), corresponding protein expression of ß-actin (2).

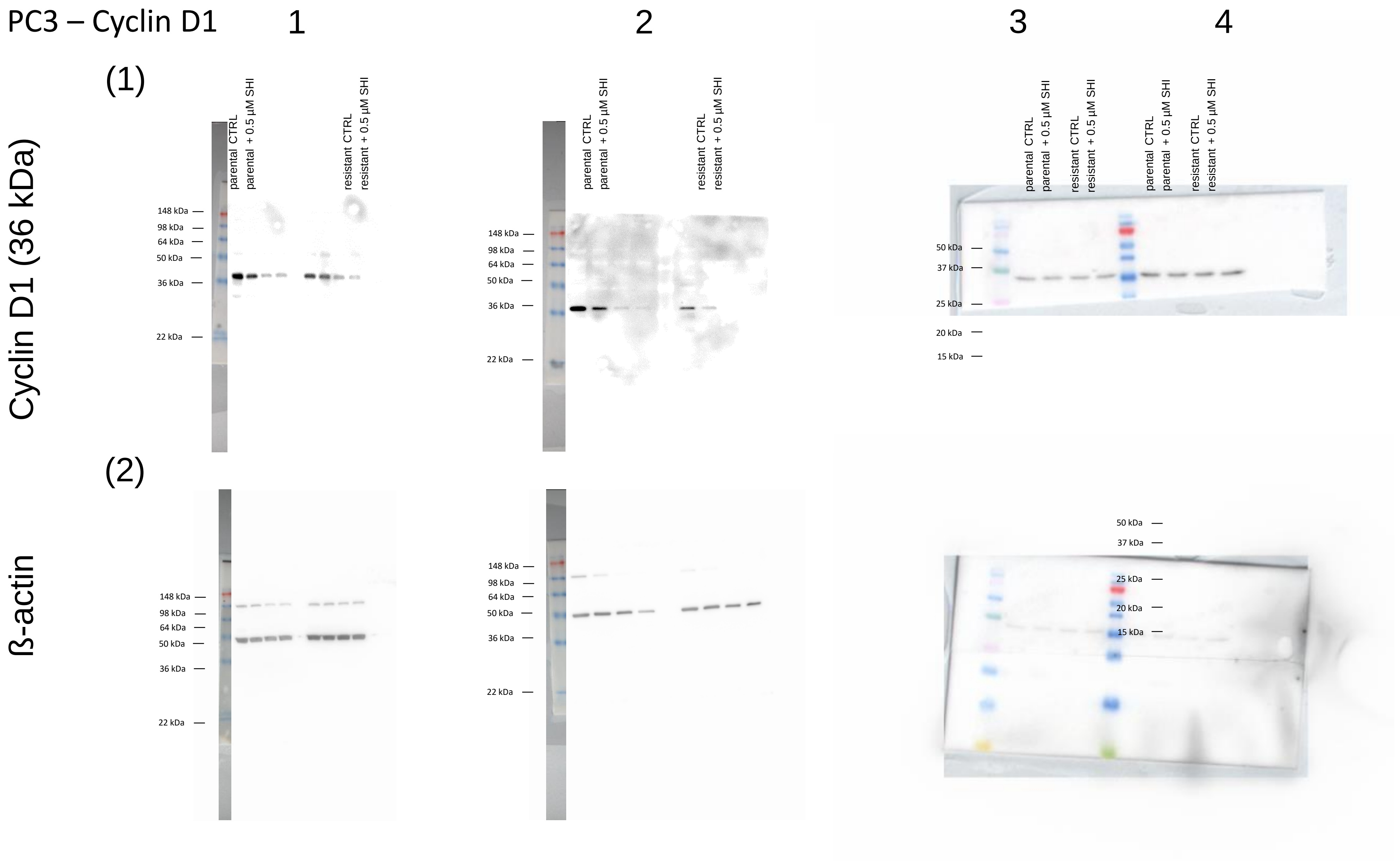

Figure S1e: Detailed information about Figure 5 - Protein expression profile of cell cycle regulating proteins in parental and DX-resistant PC3 cells. Protein expression of Cyclin D1 (1), corresponding protein expression of ß-actin (2).

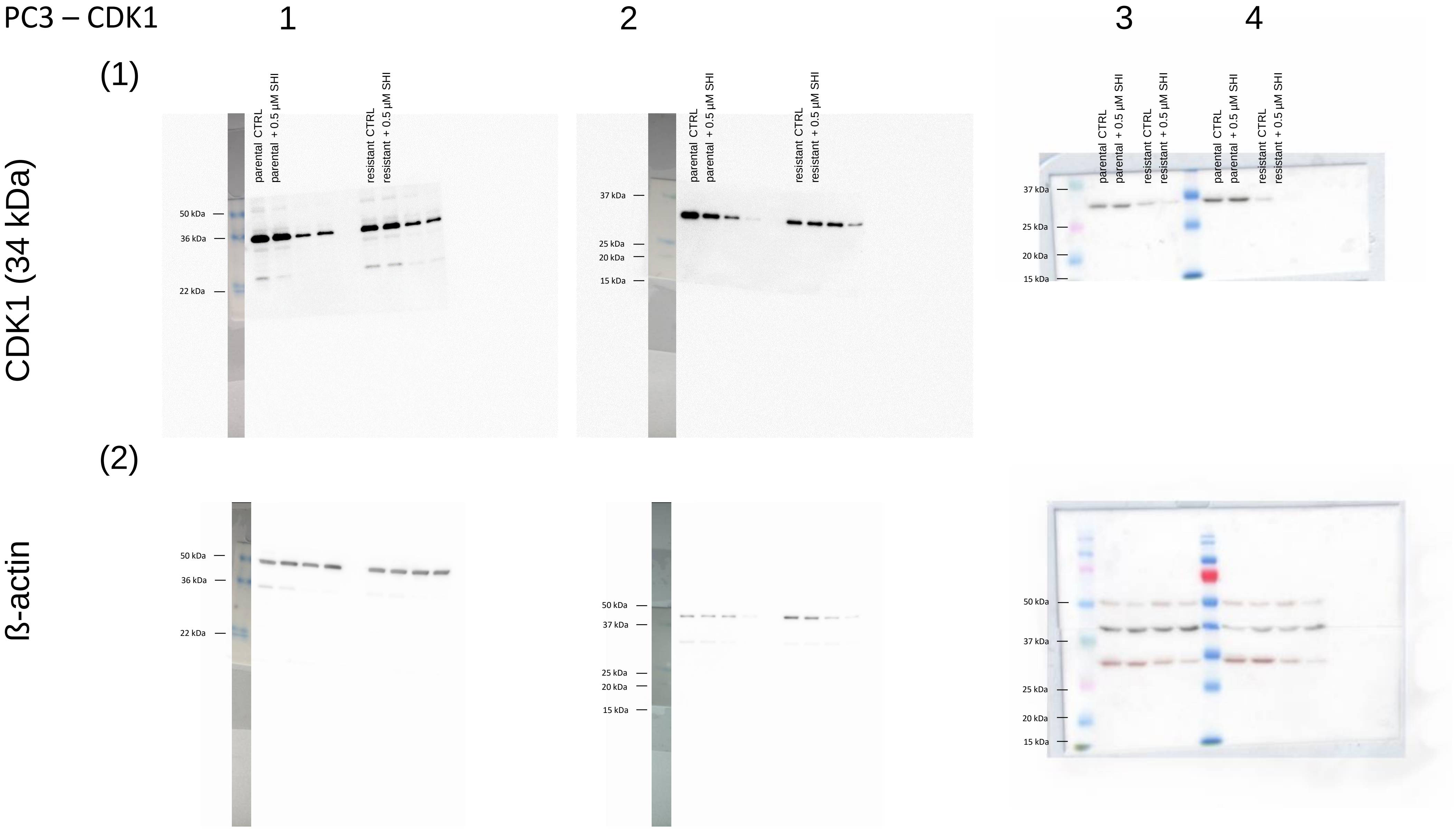

Figure S1f: Detailed information about Figure 5 - Protein expression profile of cell cycle regulating proteins in parental and DX-resistant PC3 cells. Protein expression of CDK1 (1), corresponding protein expression of ß-actin (2).

PC3 – CDK2

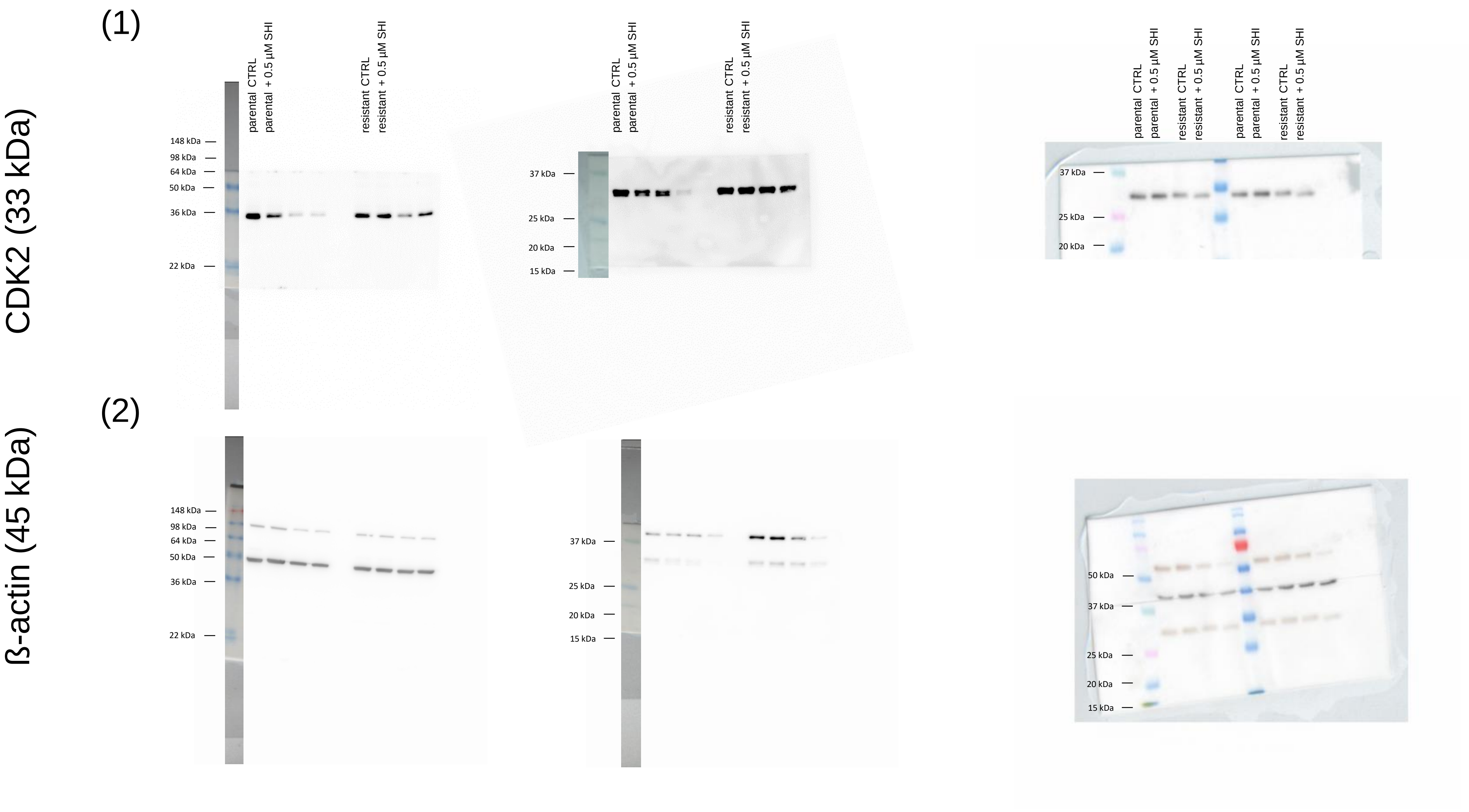

Figure S1g: Detailed information about Figure 5 - Protein expression profile of cell cycle regulating proteins in parental and DX-resistant PC3 cells. Protein expression of CDK2 (1), corresponding protein expression of ß-actin (2).

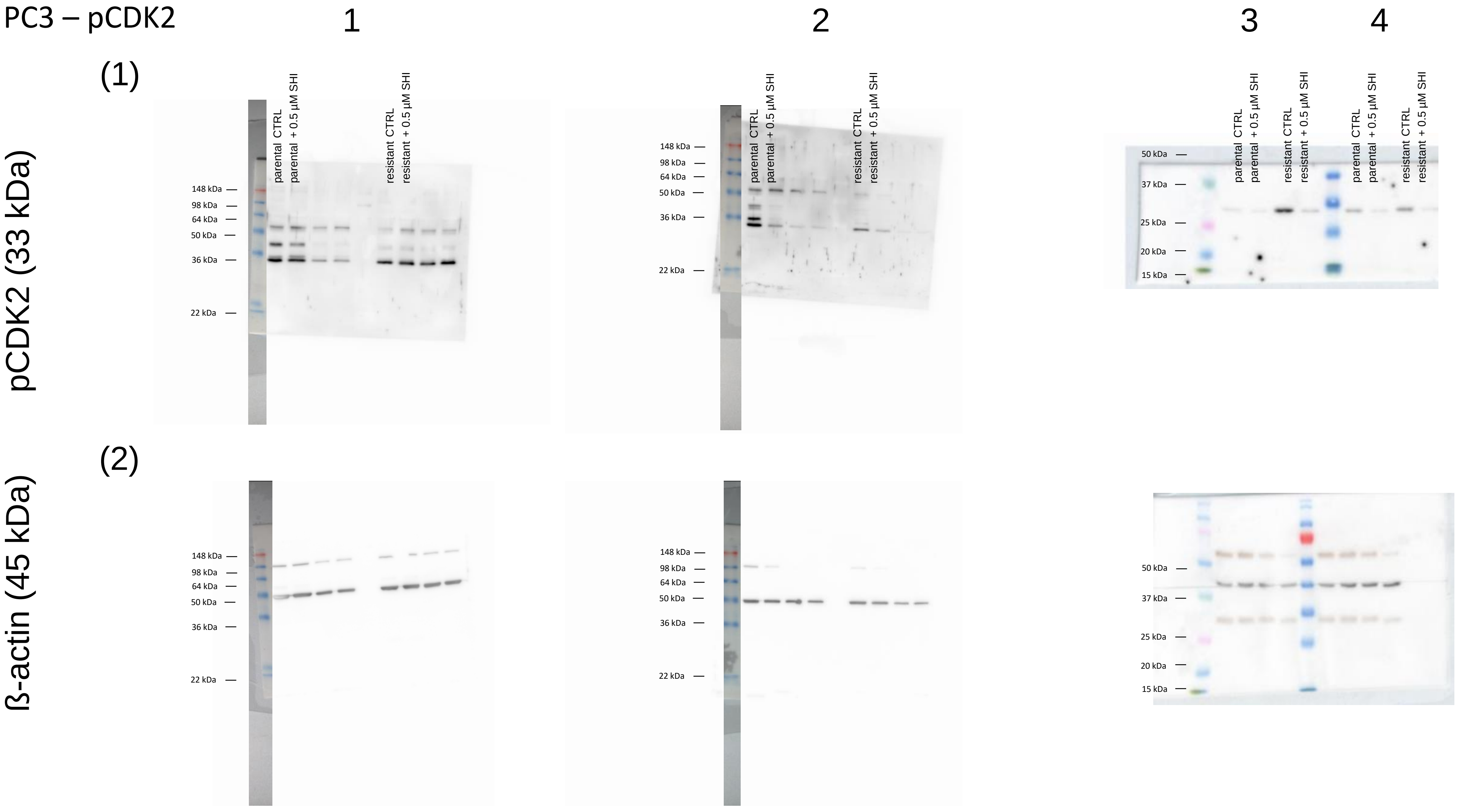

Figure S1h: Detailed information about Figure 5 - Protein expression profile of cell cycle regulating proteins in parental and DX-resistant PC3 cells. Protein expression of pCDK2 (1), corresponding protein expression of ß-actin (2).

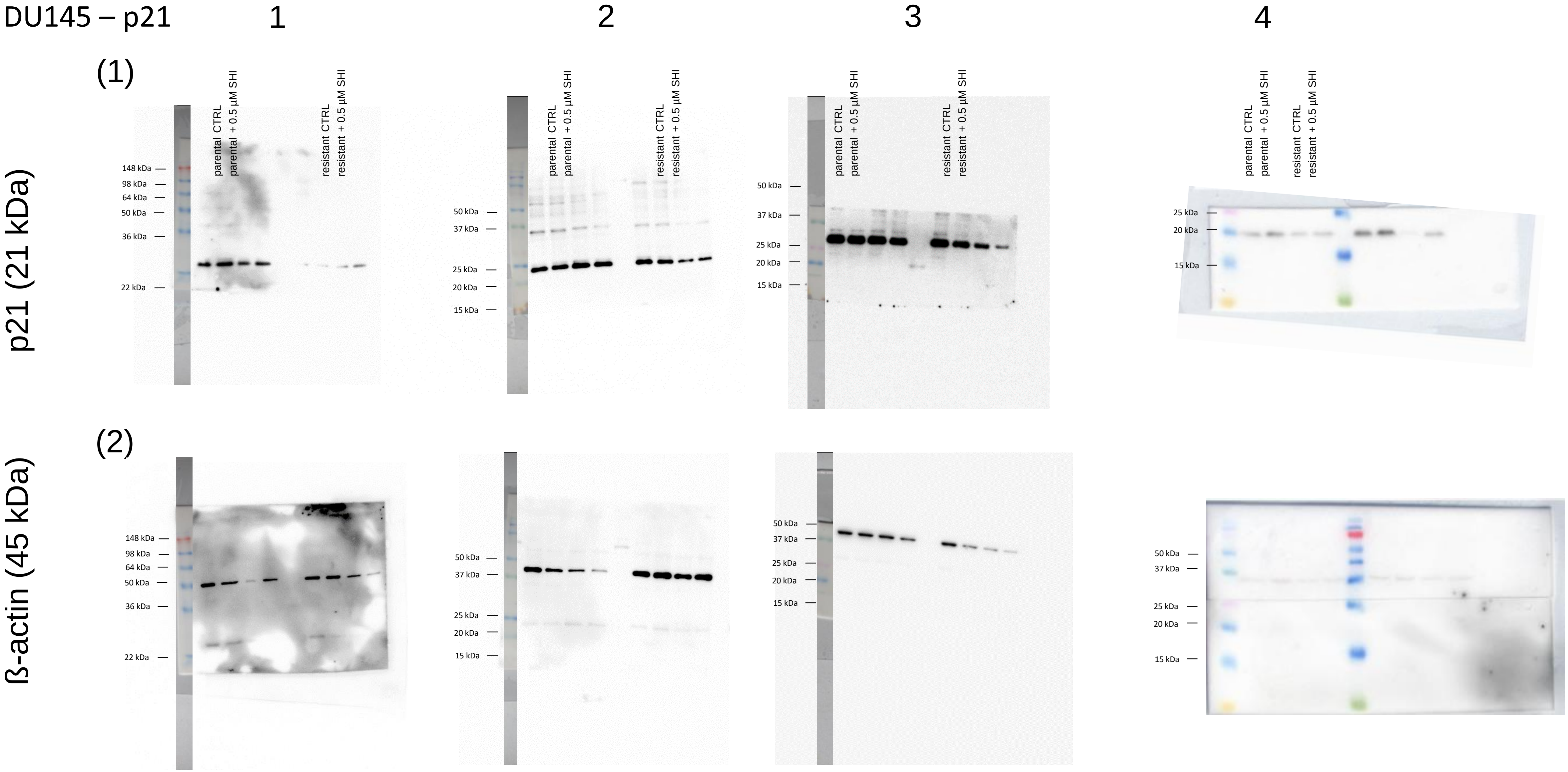

Figure S2a: Detailed information about Figure 6 - Protein expression profile of cell cycle regulating proteins in parental and DX-resistant DU145 cells. Protein expression of p21 (1), corresponding protein expression of ß-actin (2).

DU145 – p27

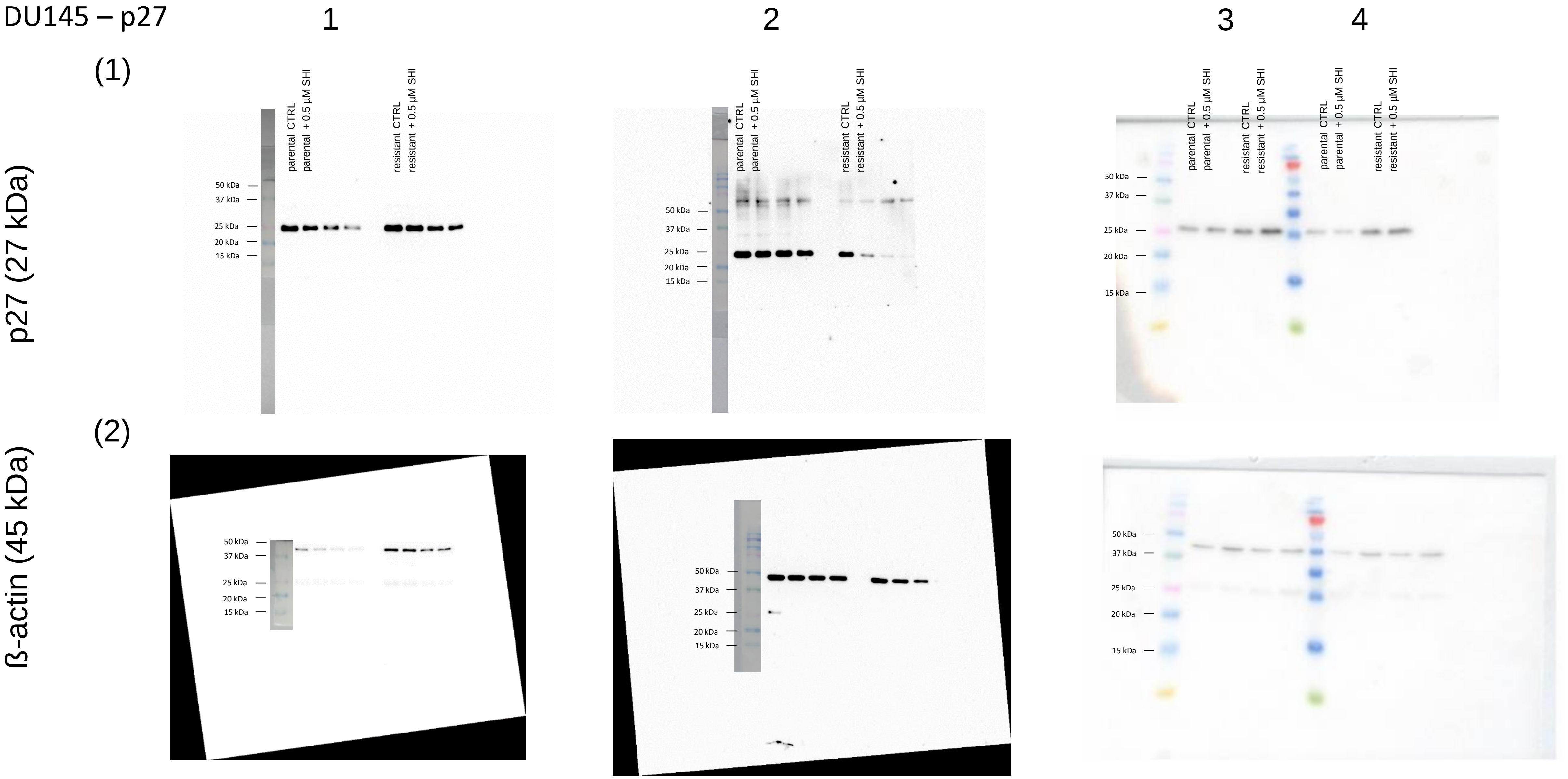

Figure S2b: Detailed information about Figure 6 - Protein expression profile of cell cycle regulating proteins in parental and DX-resistant DU145 cells. Protein expression of p27 (1), corresponding protein expression of ß-actin (2).

DU145 – Cyclin A

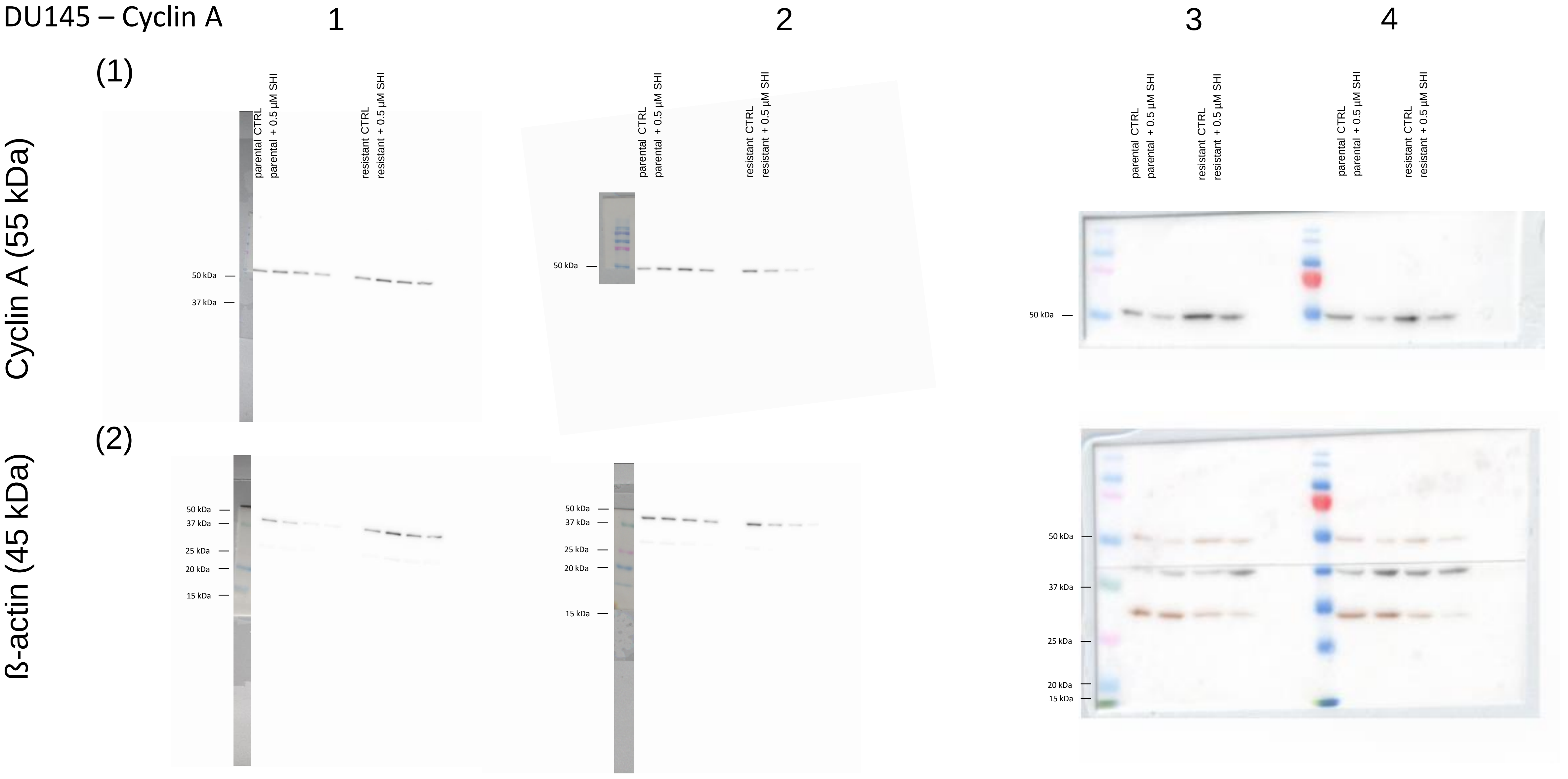

Figure S2c: Detailed information about Figure 6 - Protein expression profile of cell cycle regulating proteins in parental and DX-resistant DU145 cells. Protein expression of Cyclin A (1), corresponding protein expression of ß-actin (2).

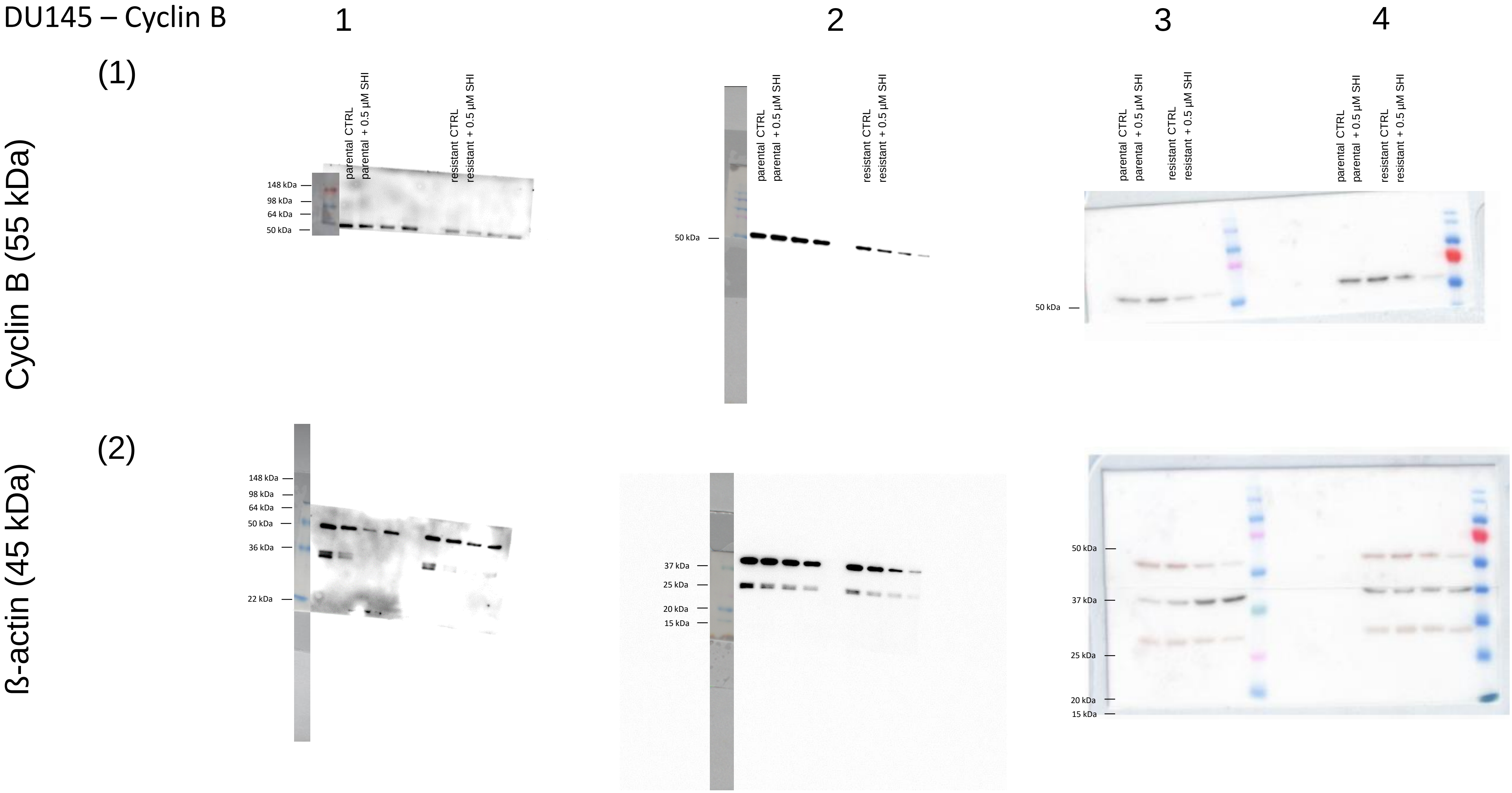

Figure S2d: Detailed information about Figure 6 - Protein expression profile of cell cycle regulating proteins in parental and DX-resistant DU145 cells. Protein expression of Cyclin B (1), corresponding protein expression of ß-actin (2).

DU145 – Cyclin D1

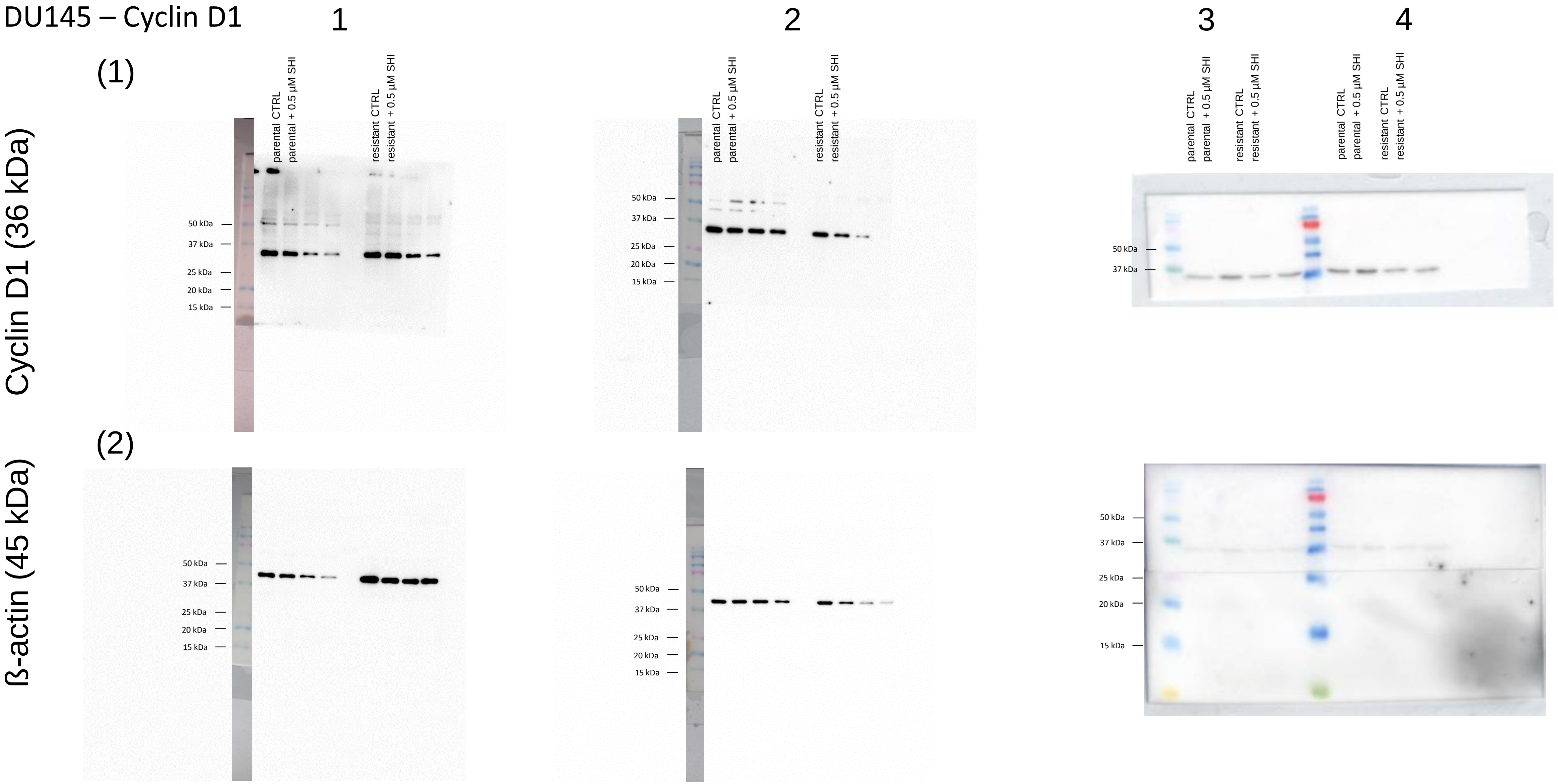

Figure S2e: Detailed information about Figure 6 - Protein expression profile of cell cycle regulating proteins in parental and DX-resistant DU145 cells. Protein expression of Cyclin D1 (1), corresponding protein expression of ß-actin (2).

DU145 – CDK1

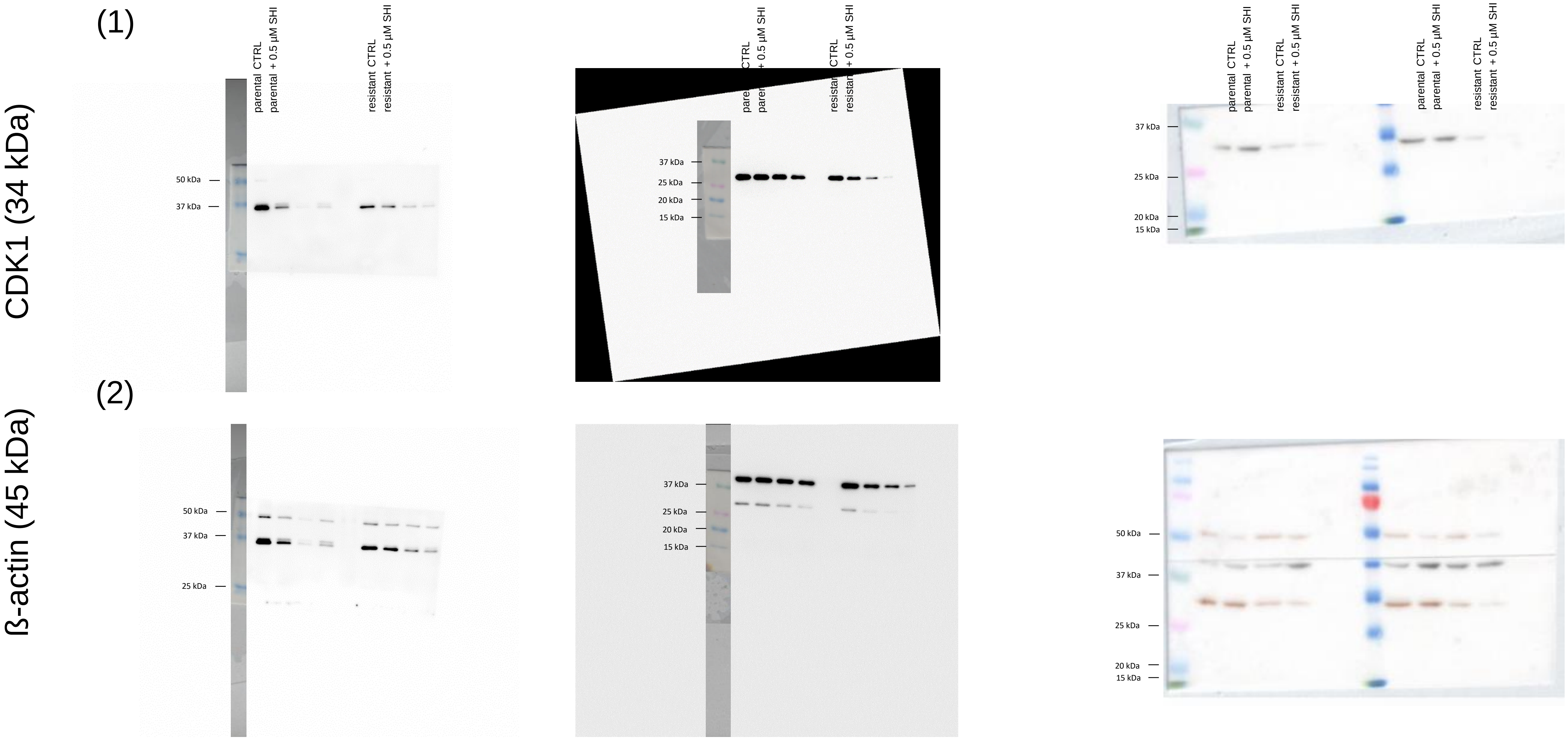

Figure S2f: Detailed information about Figure 6 - Protein expression profile of cell cycle regulating proteins in parental and DX-resistant DU145 cells. Protein expression of CDK1 (1), corresponding protein expression of ß-actin (2).

DU145 – CDK2

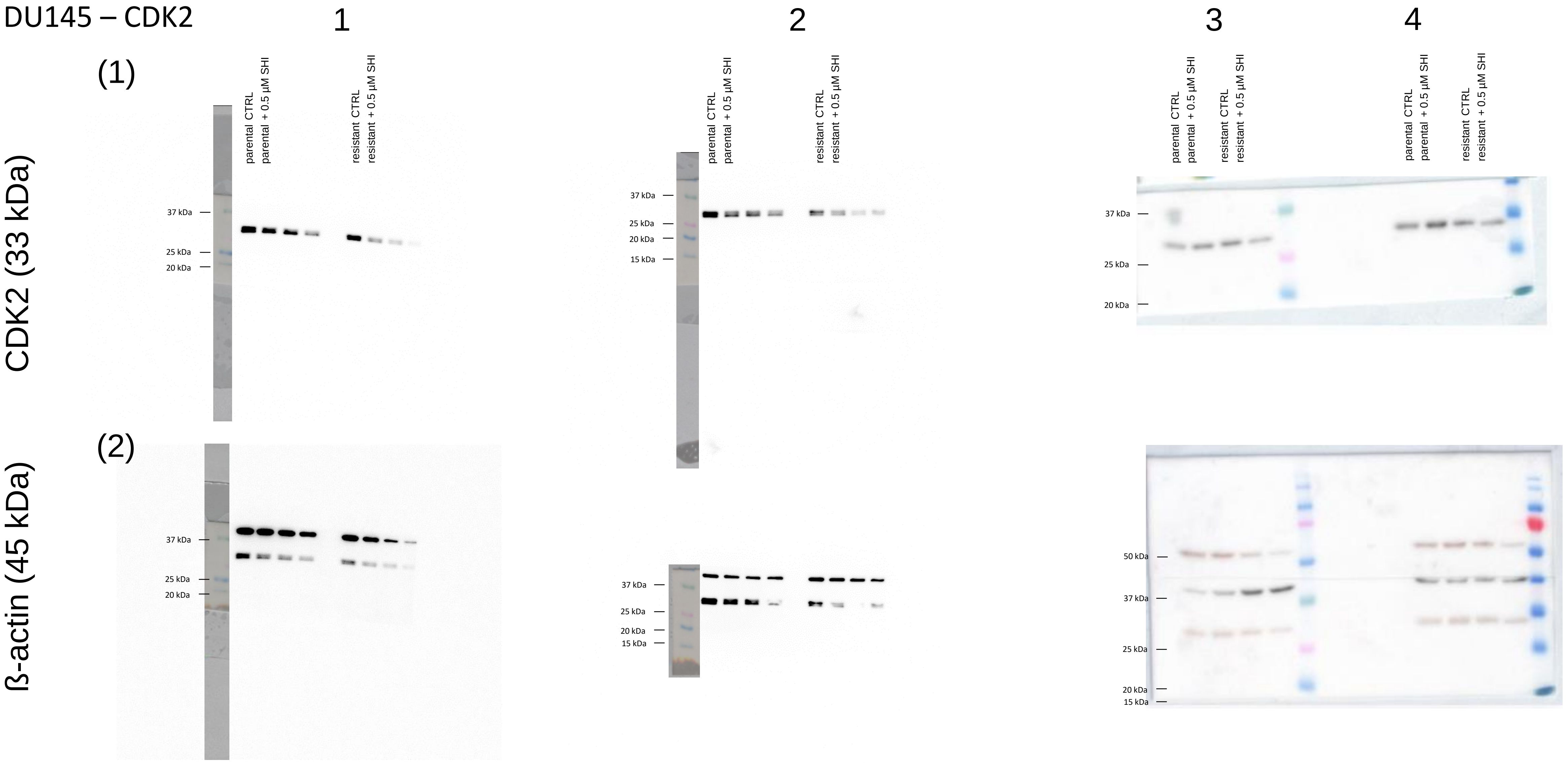

Figure S2g: Detailed information about Figure 6 - Protein expression profile of cell cycle regulating proteins in parental and DX-resistant DU145 cells. Protein expression of CDK2 (1), corresponding protein expression of ß-actin (2).

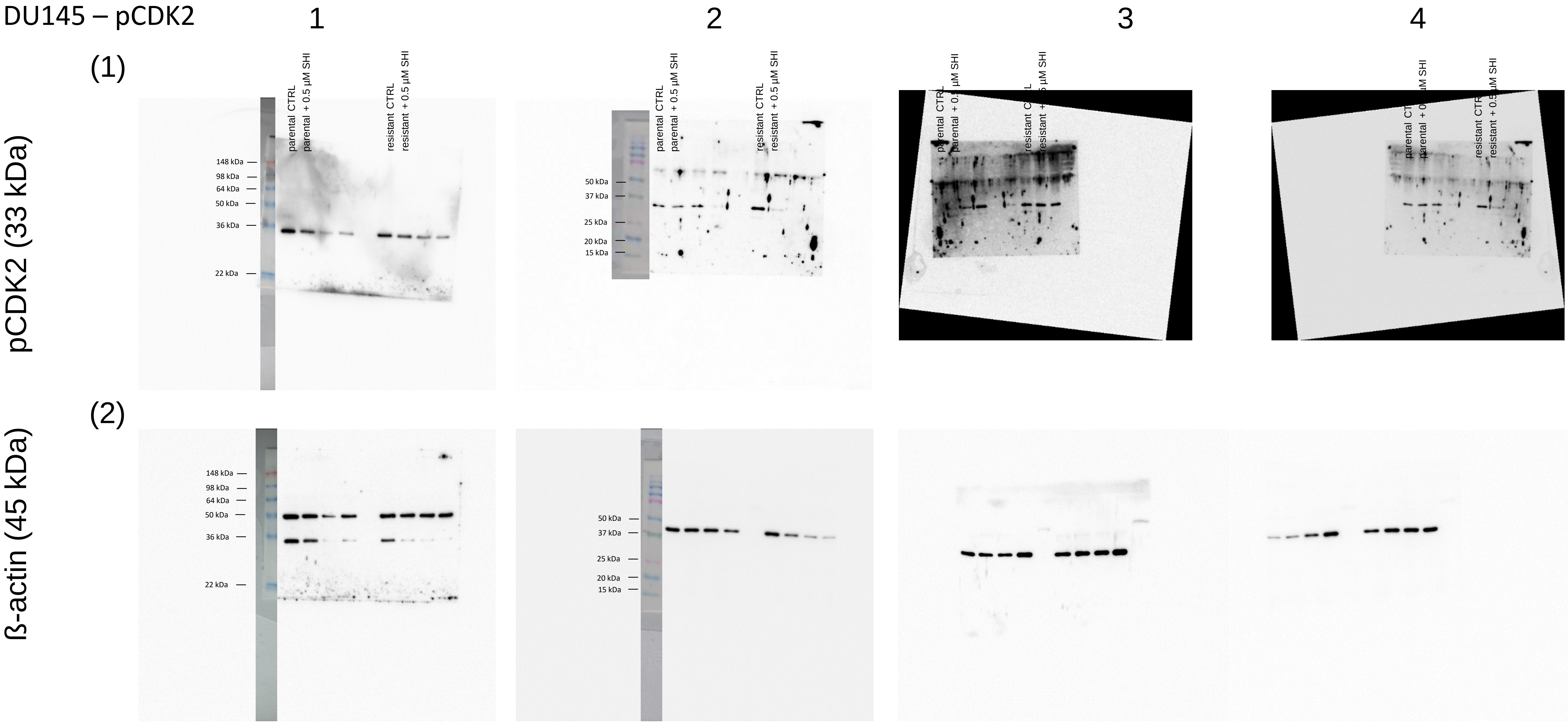

Figure S2h: Detailed information about Figure 6 - Protein expression profile of cell cycle regulating proteins in parental and DX-resistant DU145 cells. Protein expression of pCDK2 (1), corresponding protein expression of ß-actin (2).

DU145 – PARP

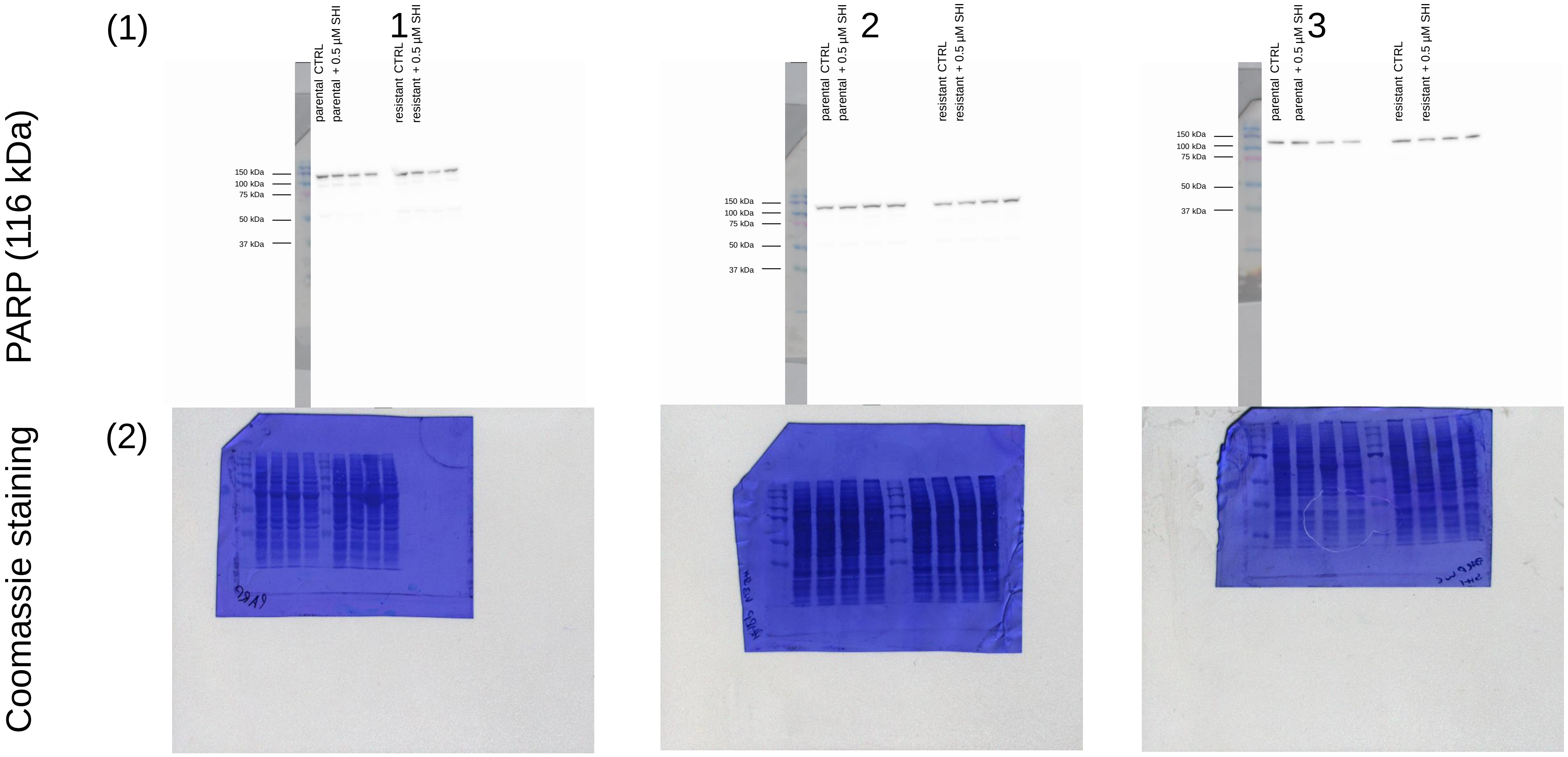

Figure S3: Detailed information about Figure 8h - Protein expression profile of PARP in parental and DX-resistant DU145 cells. Protein expression of PARP (1), corresponding Coomassie blue staining of total protein (2).

DU145 – Caspase 3

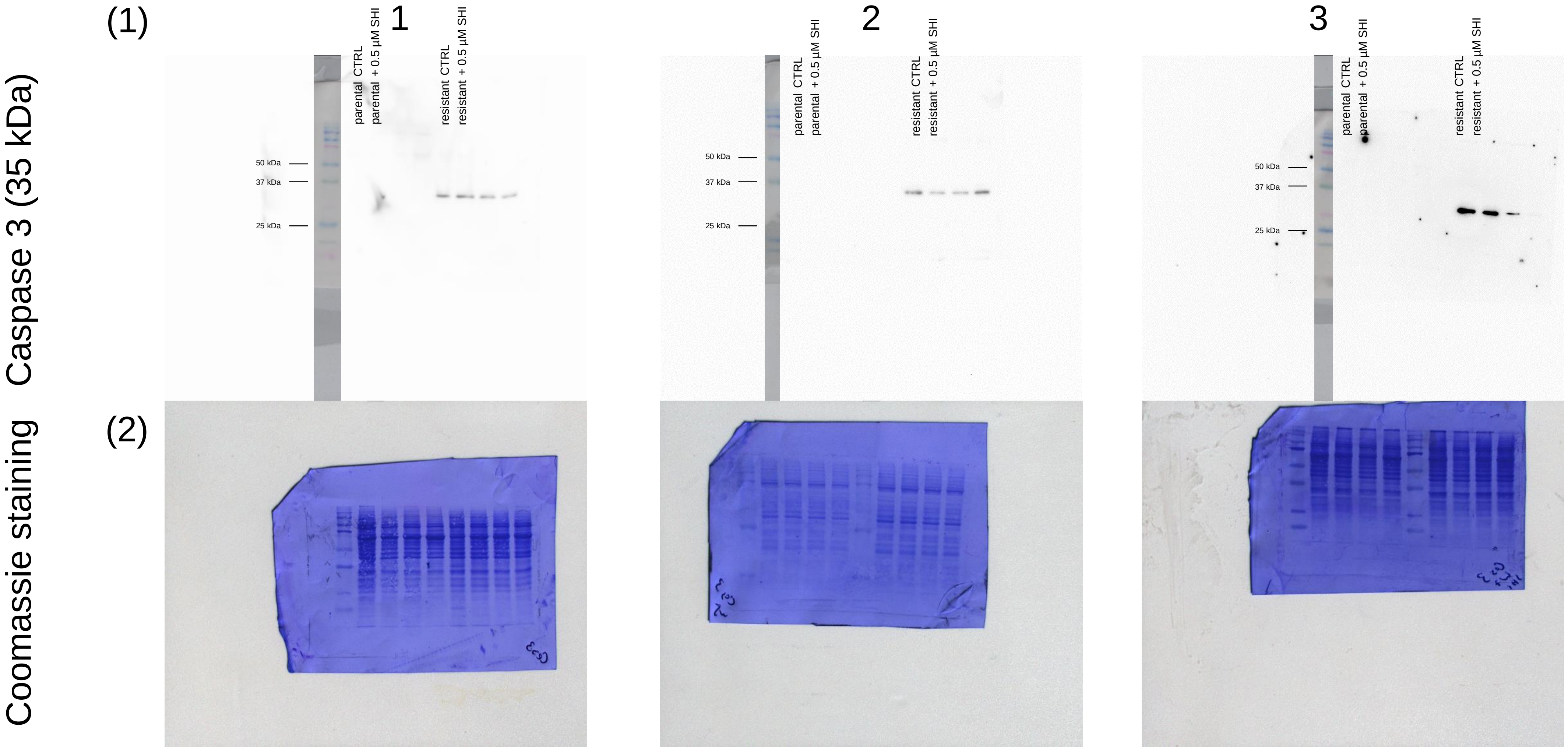

Figure S4: Detailed information about Figure 8i - Protein expression profile of Caspase 3 in parental and DX-resistant DU145 cells. Protein expression of Caspase 3 (1), corresponding Coomassie blue staining of total protein (2).

DU145 – Caspase 8

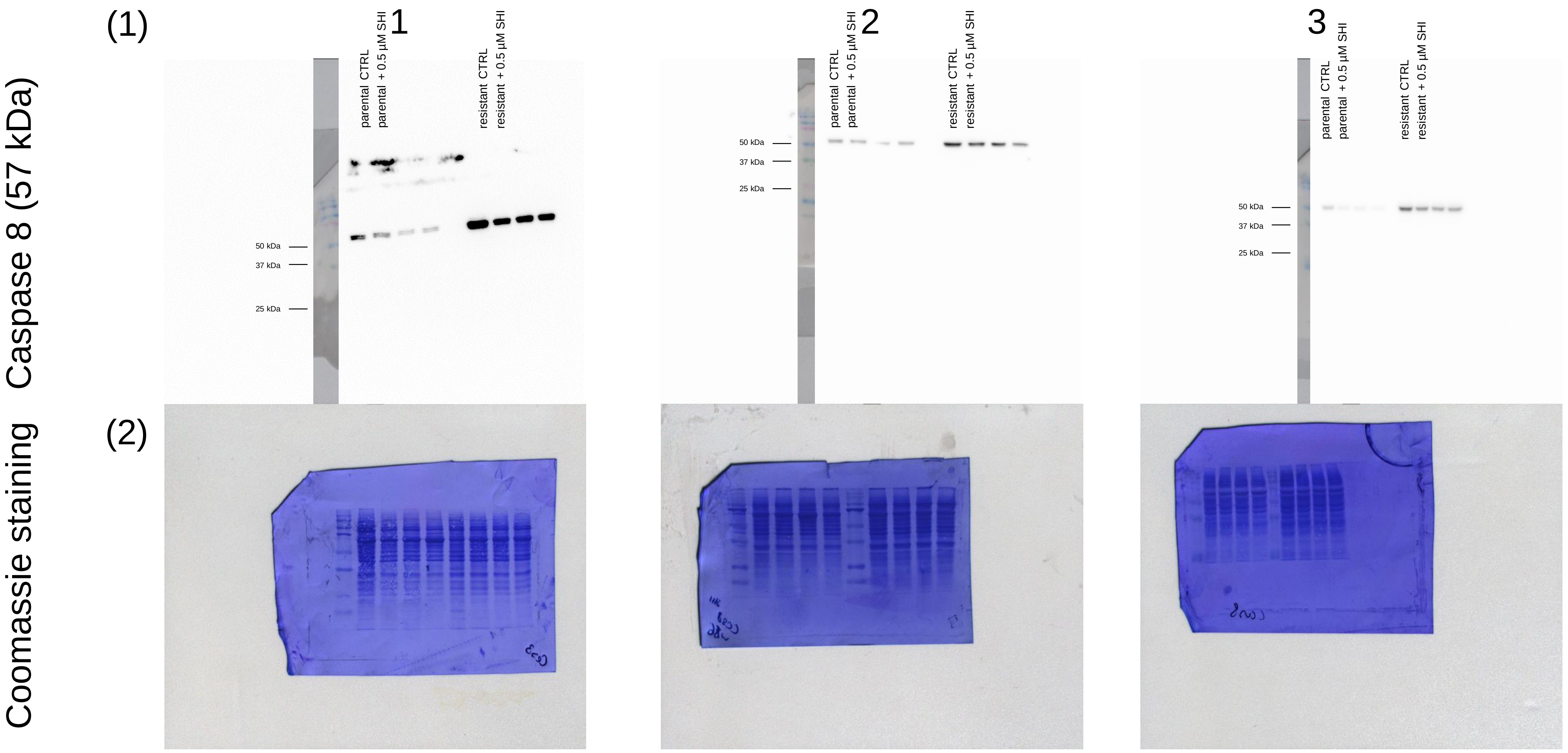

Figure S5: Detailed information about Figure 8j - Protein expression profile of Caspase 8 in parental and DX-resistant DU145 cells. Protein expression of Caspase 8 (1), corresponding Coomassie blue staining of total protein (2).

PC3 – RIP1

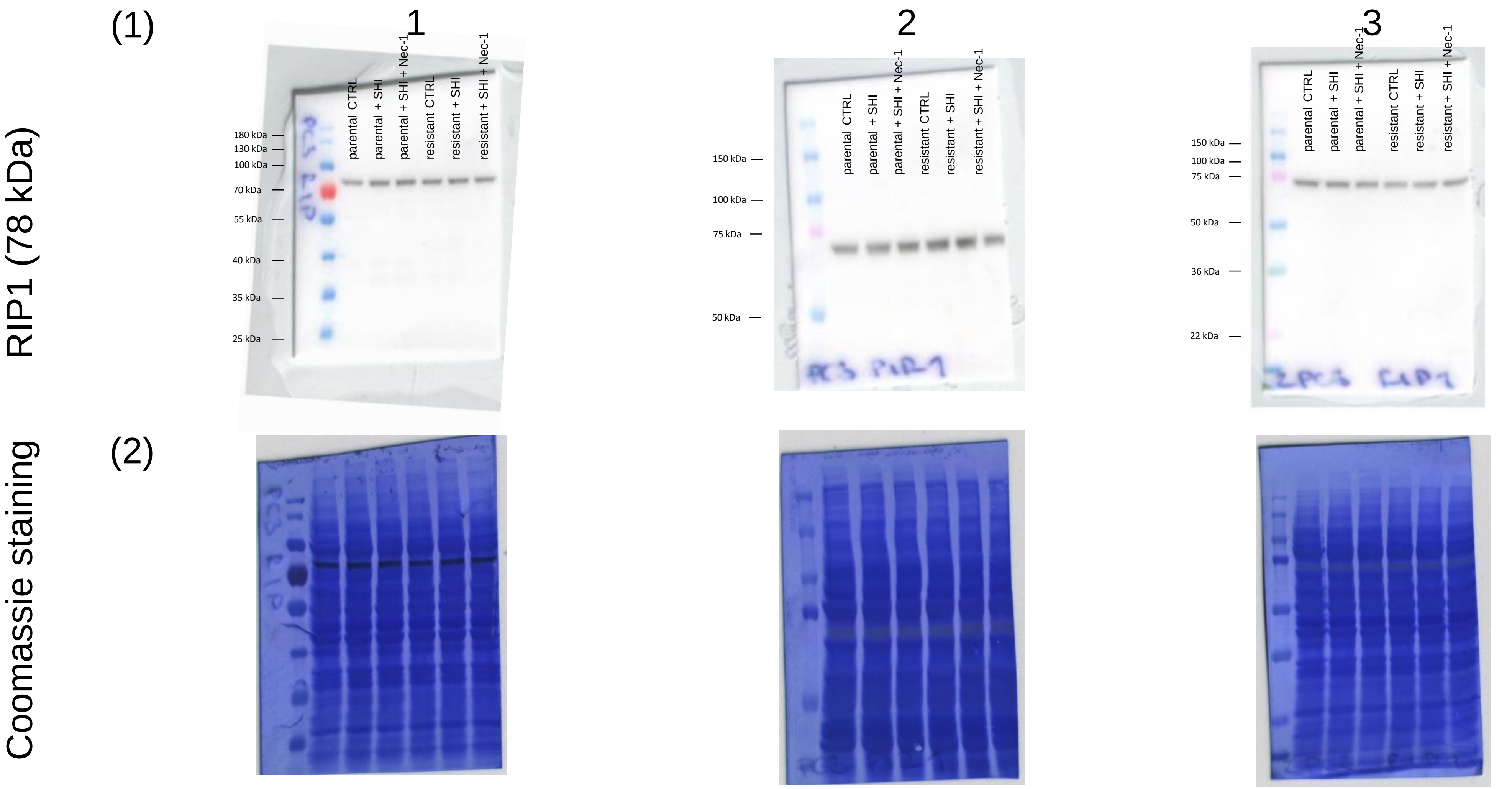

Figure S6: Detailed information about Figure 10a - Protein expression profile of RIP1 in parental and DX-resistant PC3 cells. Protein expression of RIP1 (1), corresponding Coomassie blue staining of total protein (2).

PC3 – pRIP1

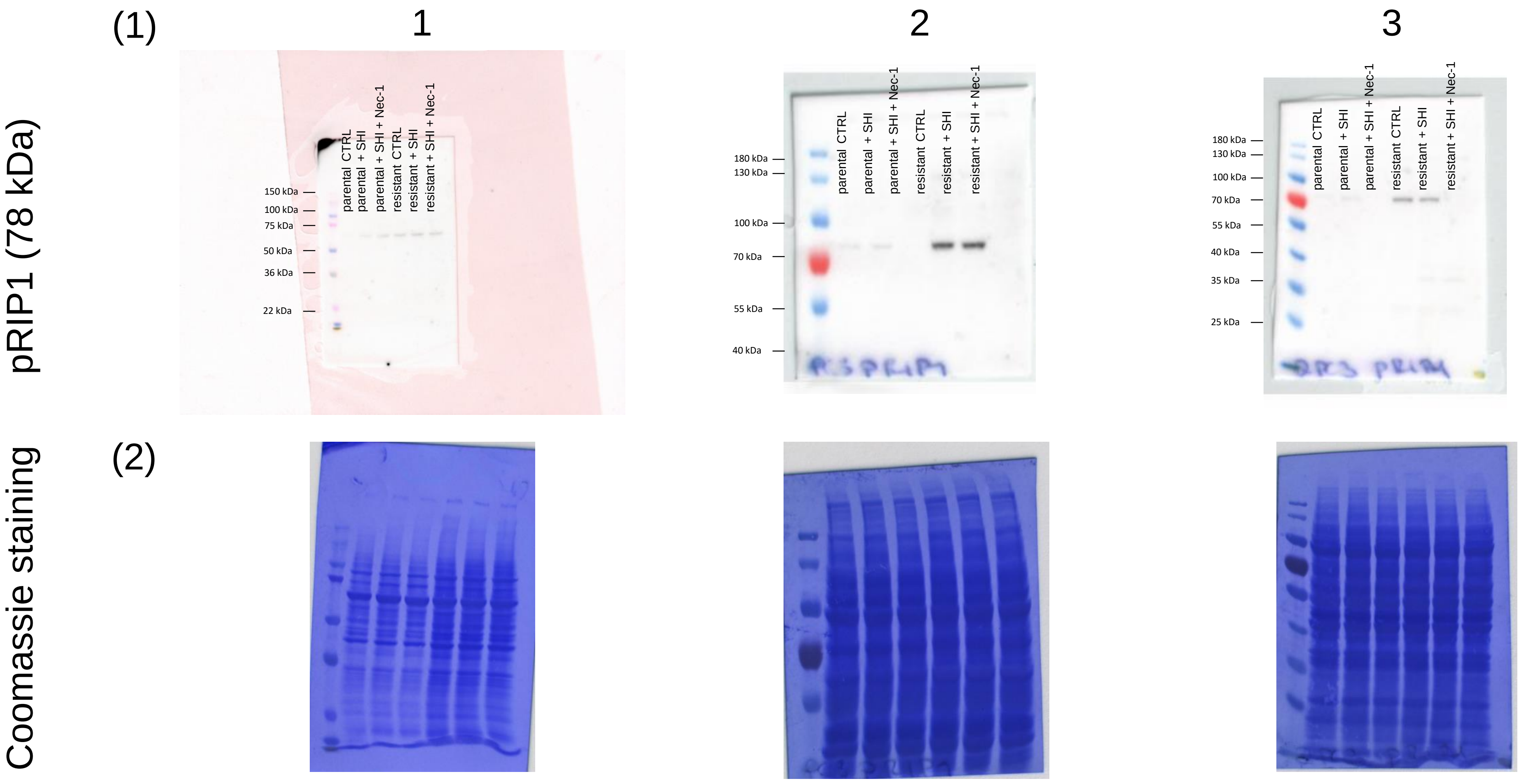

Figure S6: Detailed information about Figure 10b - Protein expression profile of pRIP1 in parental and DX-resistant PC3 cells. Protein expression of pRIP1 (1), corresponding Coomassie blue staining of total protein (2).

PC3 – RIP3

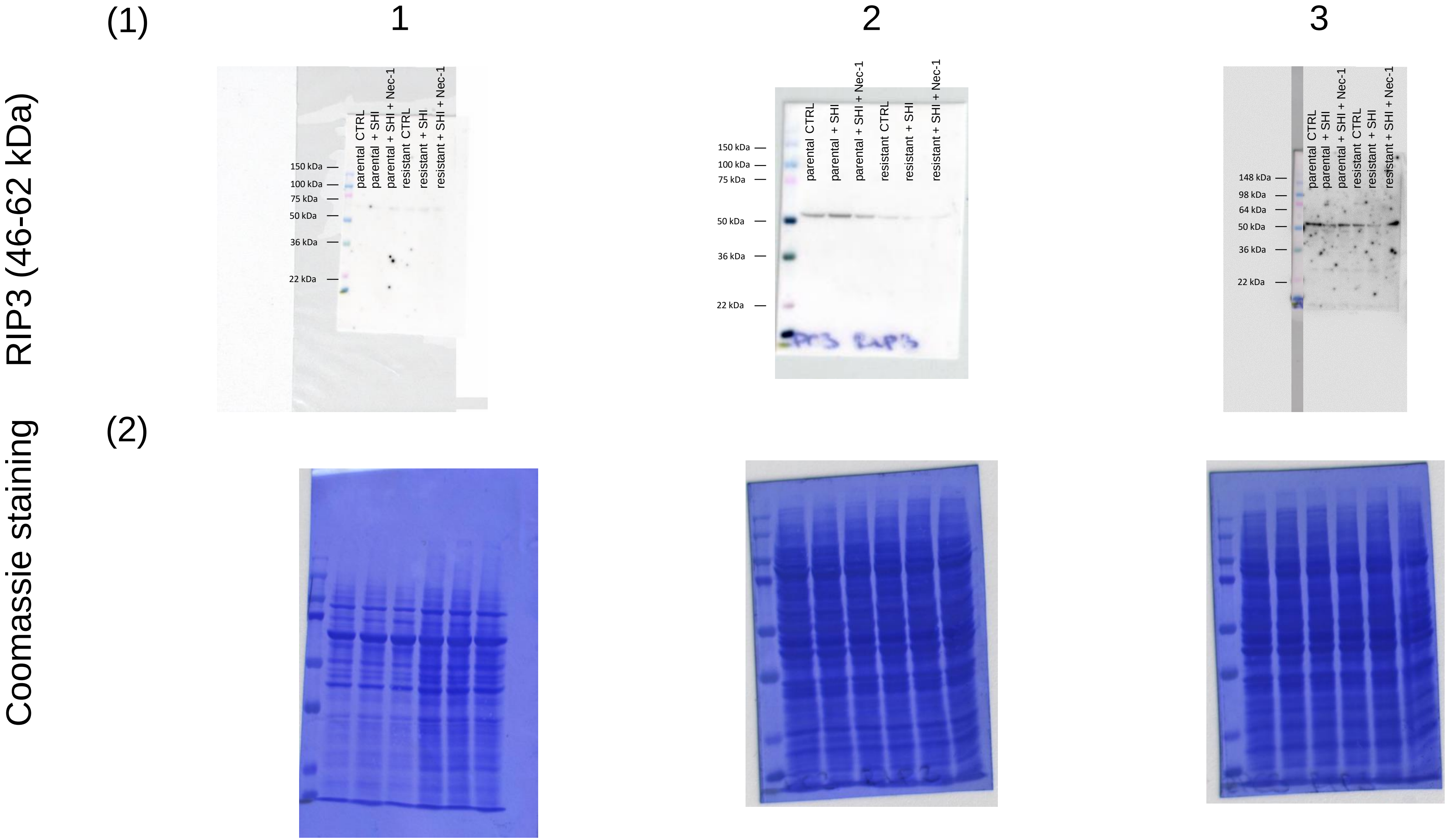

Figure S6: Detailed information about Figure 10c - Protein expression profile of RIP3 in parental and DX-resistant PC3 cells. Protein expression of RIP3 (1), corresponding Coomassie blue staining of total protein (2).

PC3 – pRIP3

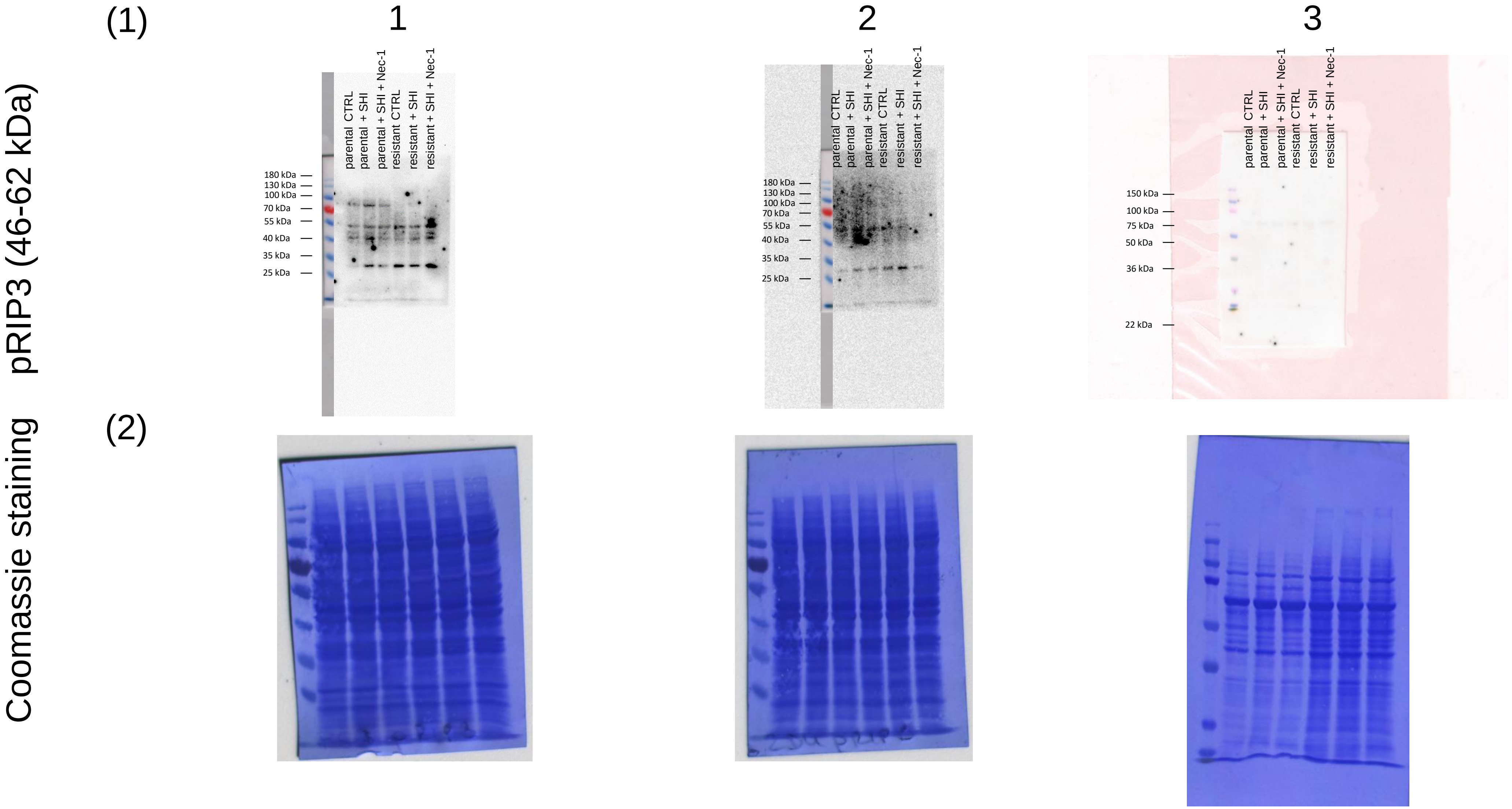

Figure S6: Detailed information about Figure 10d - Protein expression profile of pRIP3 in parental and DX-resistant PC3 cells. Protein expression of pRIP3 (1), corresponding Coomassie blue staining of total protein (2).

PC3 – MLKL

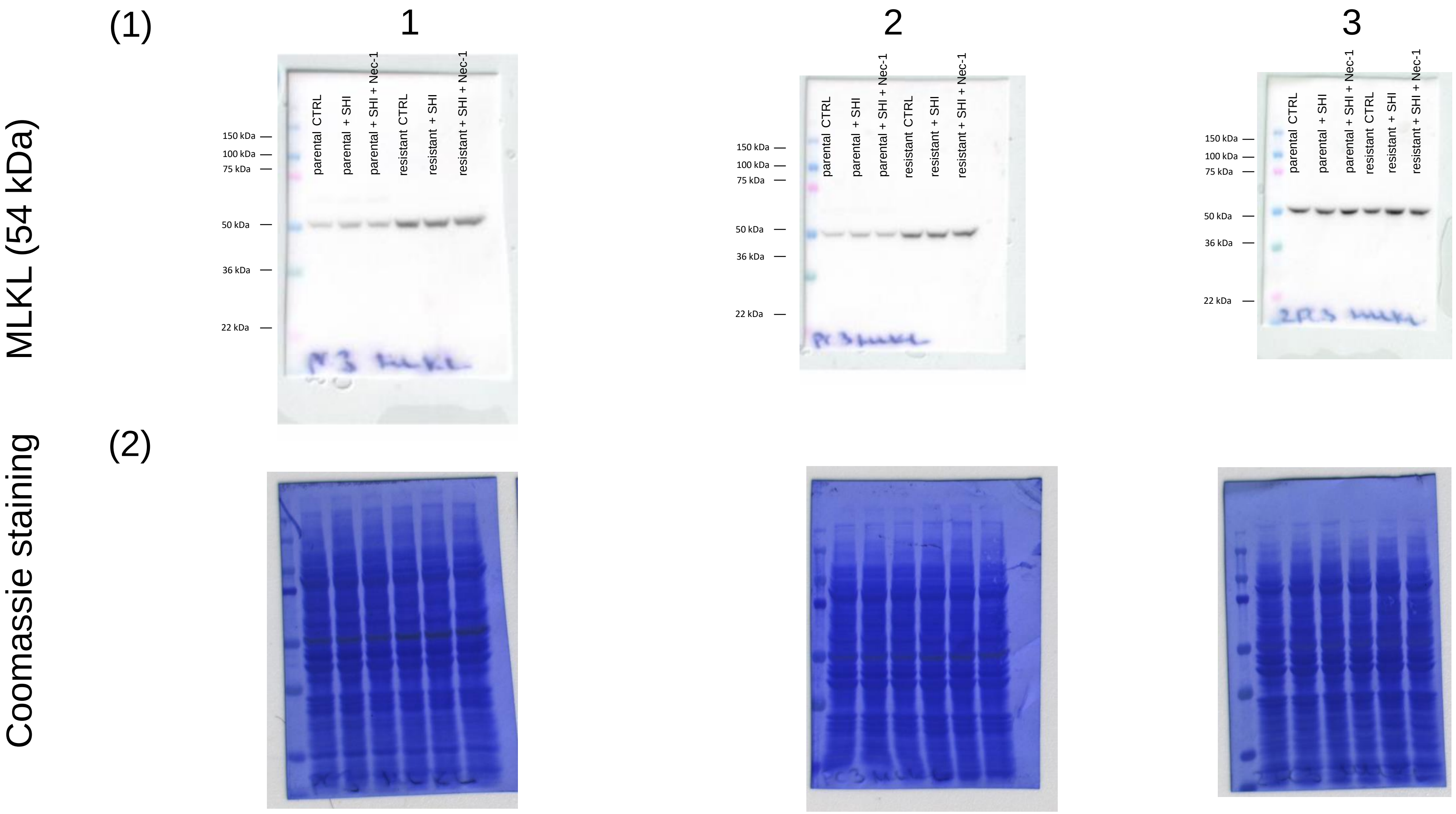

Figure S6: Detailed information about Figure 10e - Protein expression profile of MLKL in parental and DX-resistant PC3 cells. Protein expression of MLKL (1), corresponding Coomassie blue staining of total protein (2).

DU145 – RIP1

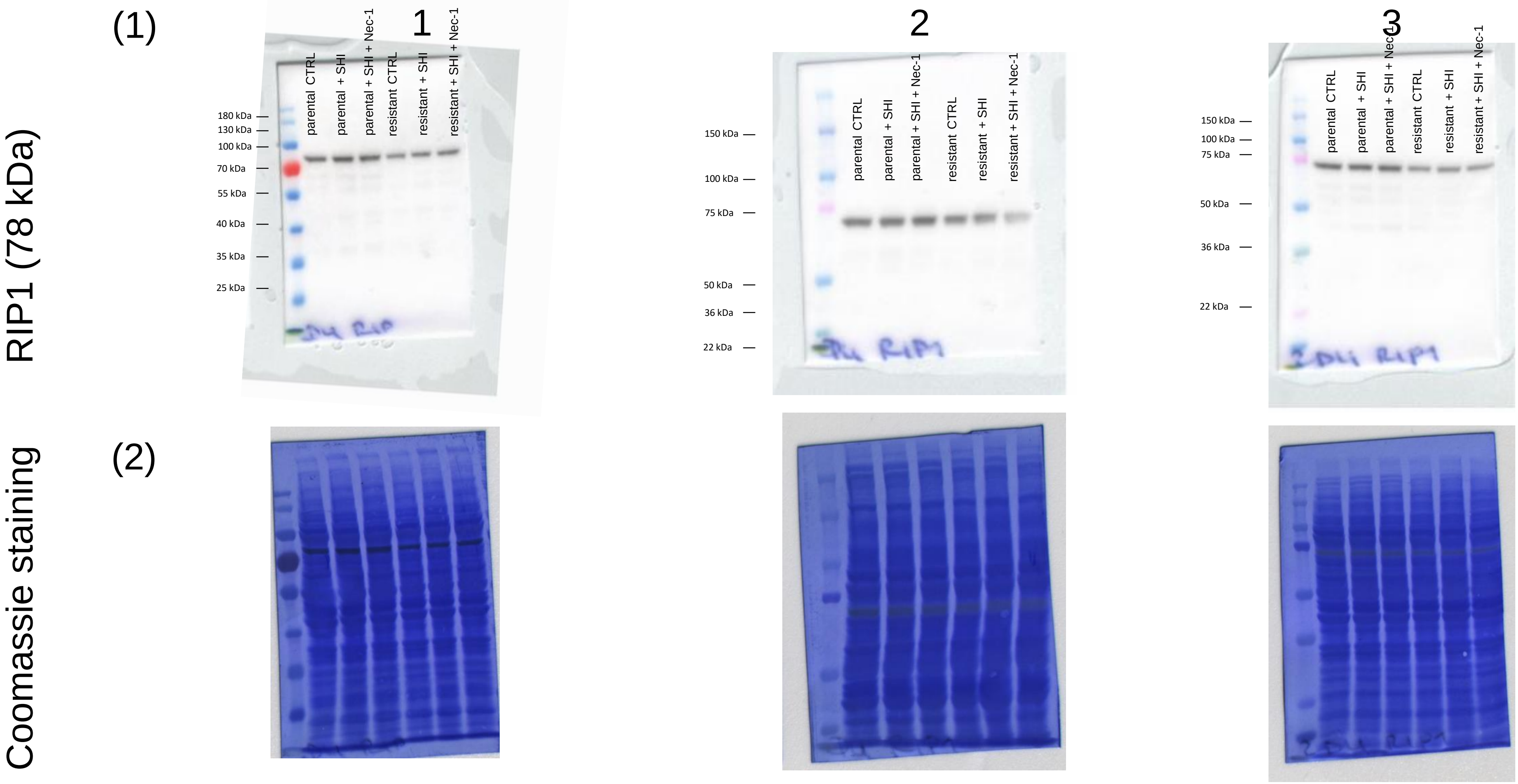

Figure S6: Detailed information about Figure 10f - Protein expression profile of RIP1 in parental and DX-resistant DU145 cells. Protein expression of RIP1 (1), corresponding Coomassie blue staining of total protein (2).

DU145 – pRIP1

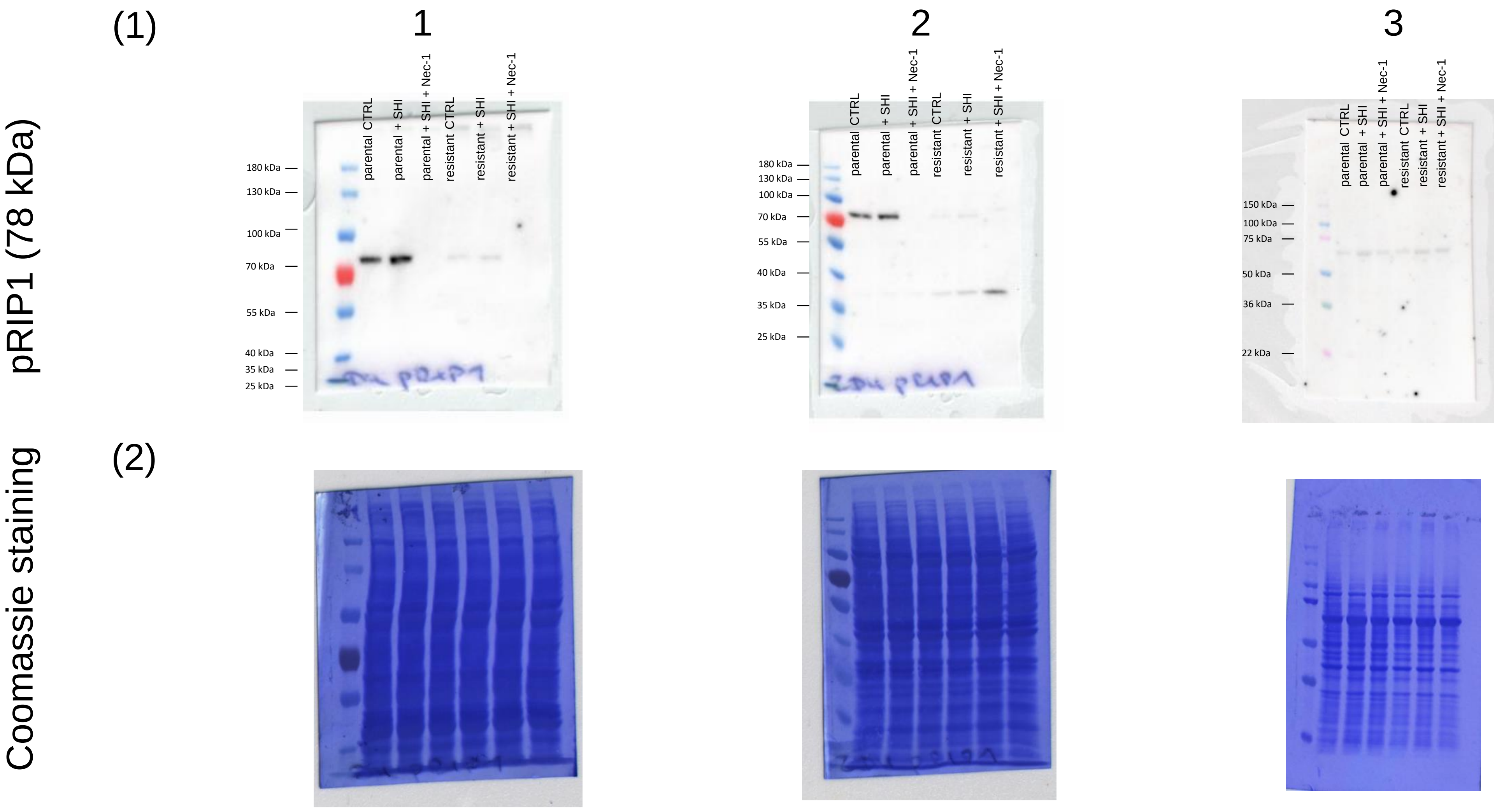

Figure S6: Detailed information about Figure 10g - Protein expression profile of pRIP1 in parental and DX-resistant DU145 cells. Protein expression of pRIP1 (1), corresponding Coomassie blue staining of total protein (2).

DU145 – RIP3

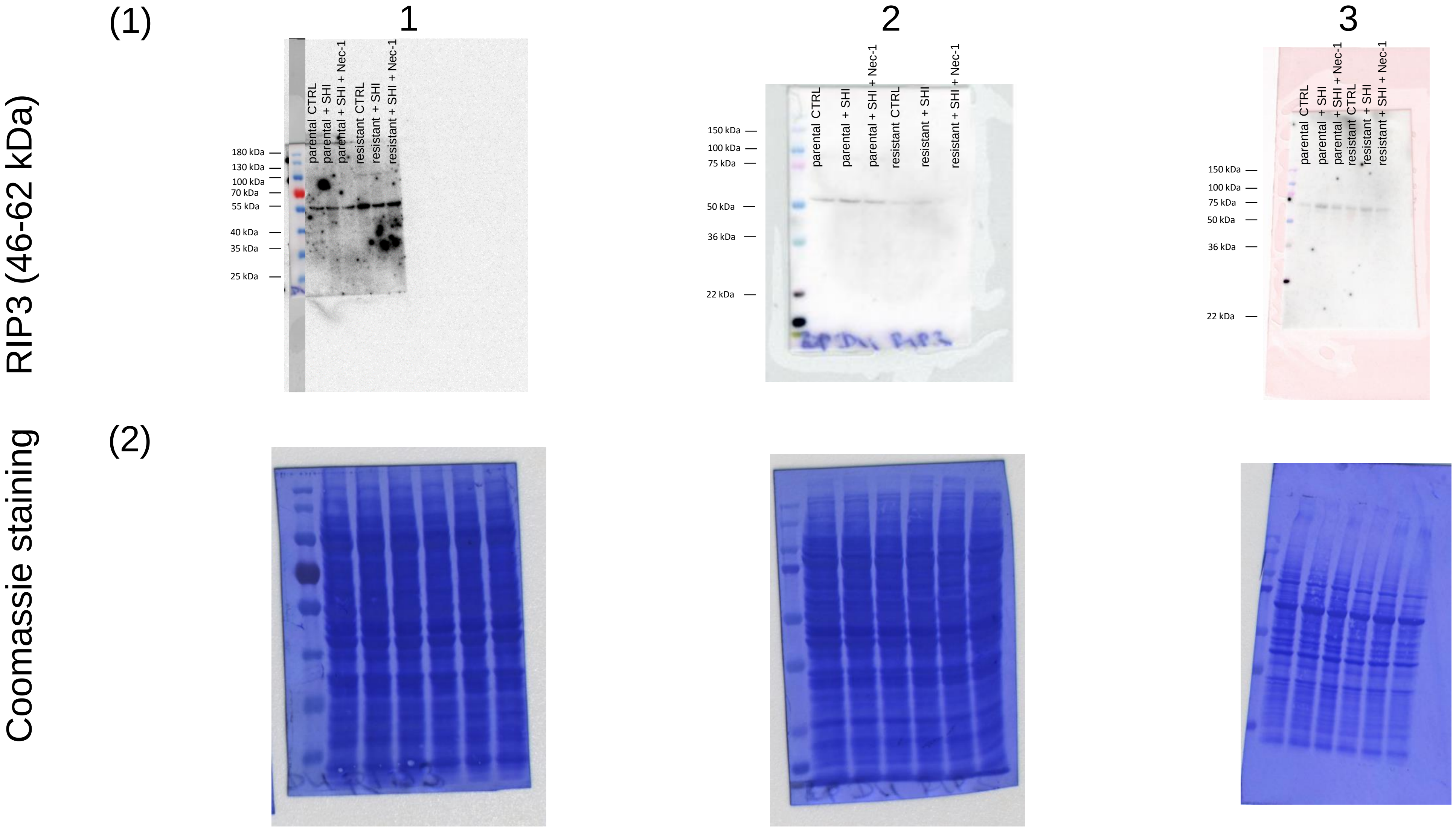

Figure S6: Detailed information about Figure 10h - Protein expression profile of RIP3 in parental and DX-resistant DU145 cells. Protein expression of RIP3 (1), corresponding Coomassie blue staining of total protein (2).

DU145 – pRIP3

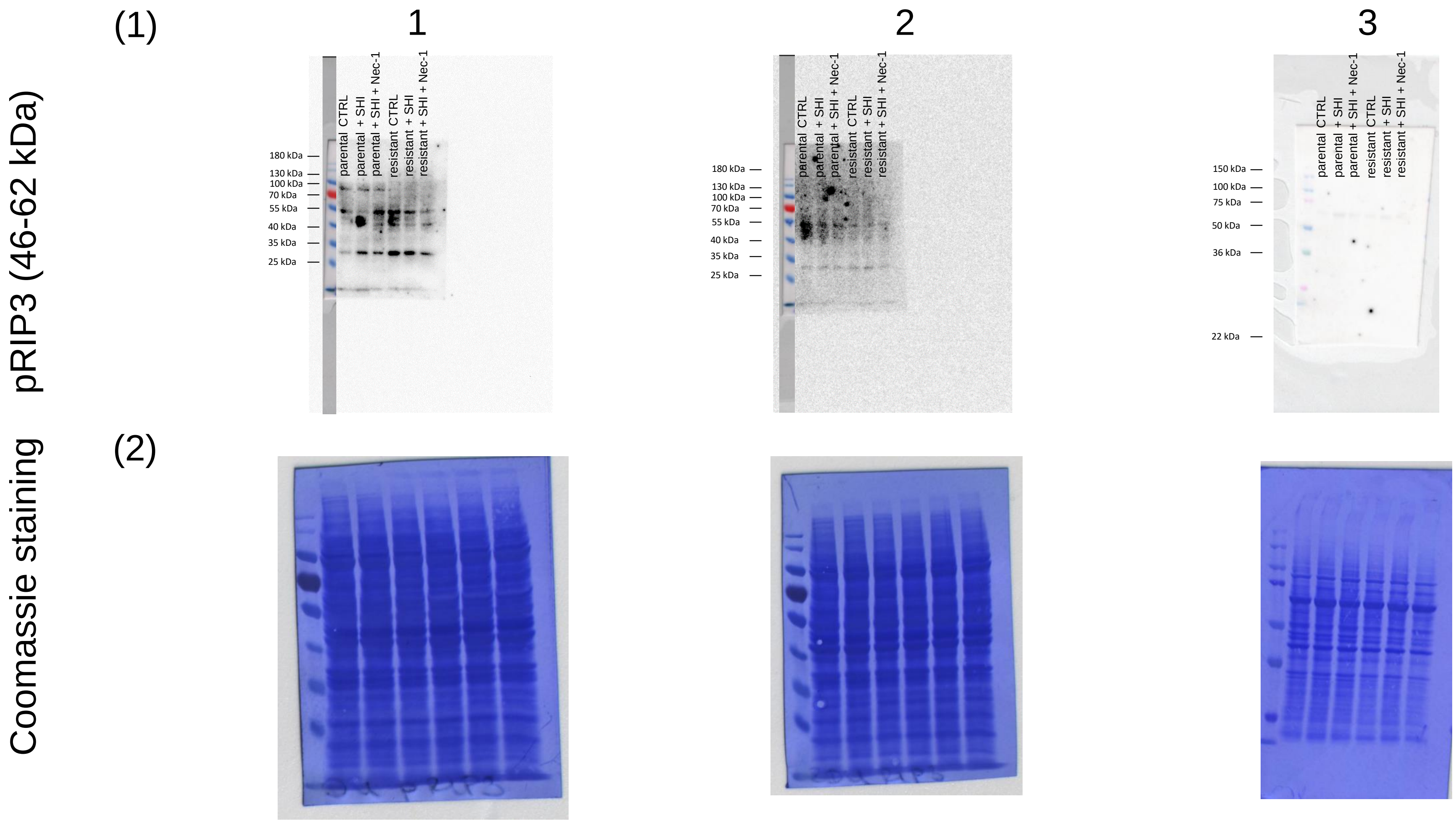

Figure S6: Detailed information about Figure 10i - Protein expression profile of pRIP3 in parental and DX-resistant DU145 cells. Protein expression of pRIP3 (1), corresponding Coomassie blue staining of total protein (2).

DU145 – MLKL

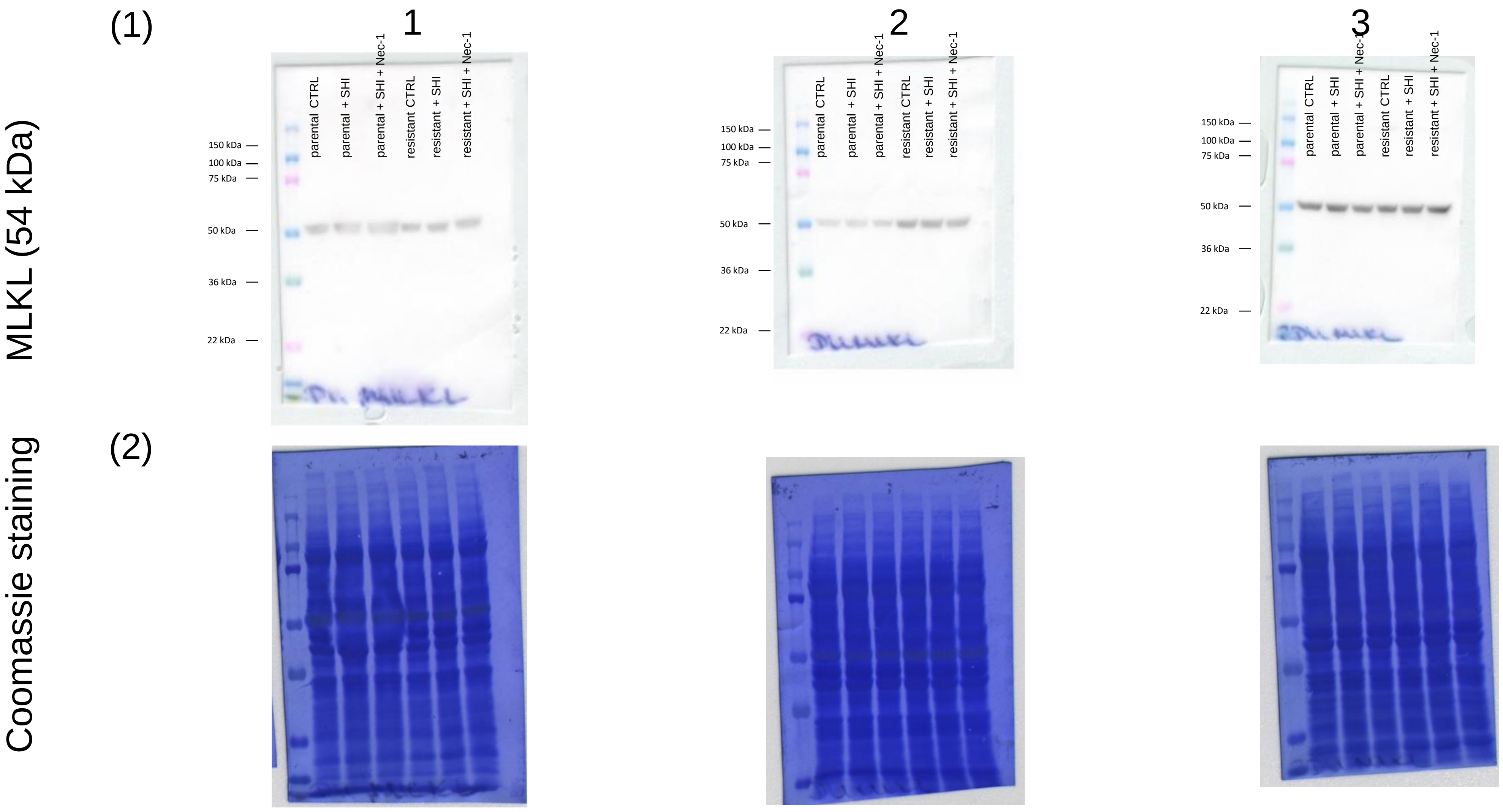

Figure S6: Detailed information about Figure 10j - Protein expression profile of MLKL in parental and DX-resistant DU145 cells. Protein expression of MLKL (1), corresponding Coomassie blue staining of total protein (2).

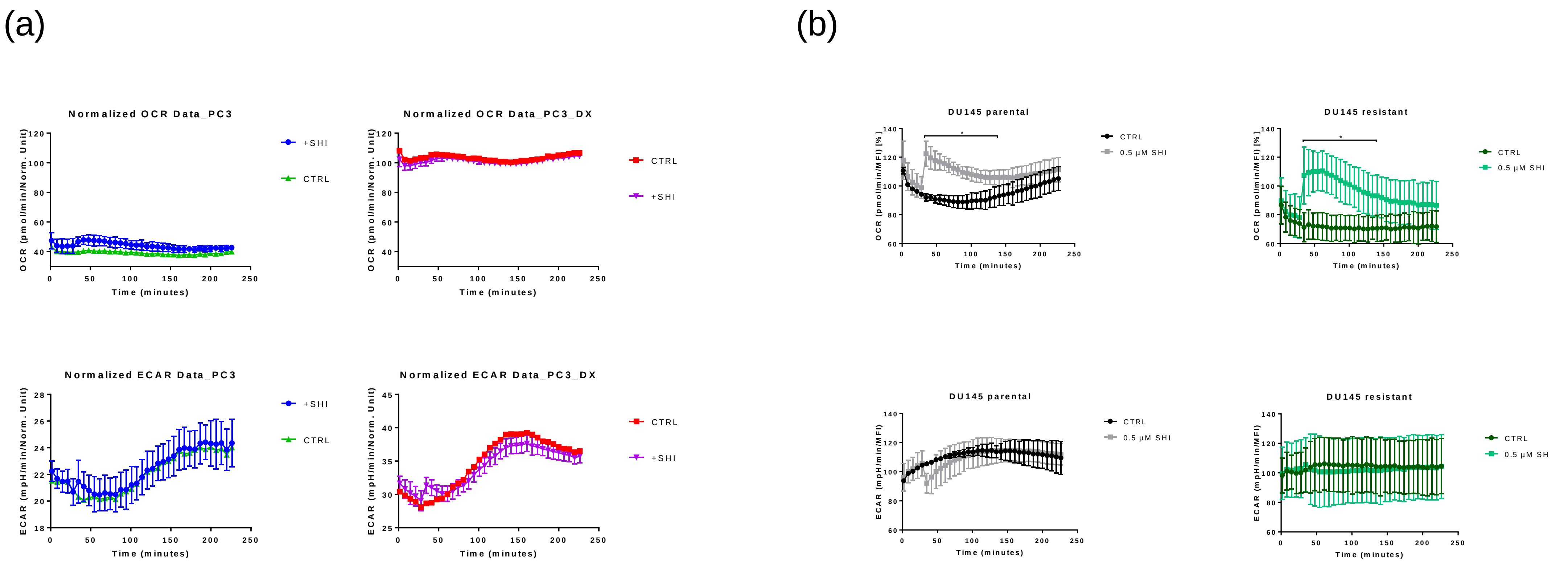

Figure S7: Detailed information about section 2.6 - Shikonin showed no effects on metabolism. Oxygen consumption rate (OCR) (top) and extracellular acidification rate (ECAR) (bottom) of PC3 and PC3_DX (a) as well as DU145 and DU145_DX (b) cells. Parental and DX-resistant cells untreated or treated with SHI 0.5 µM for 20 h (n = 3).
